# Preparing early economic evaluations for the development and management of health service interventions

**DOI:** 10.1017/S0266462324000539

**Published:** 2024-10-18

**Authors:** Andrew Partington, Maria Crotty, Kate Laver, Leanne Greene, Hossein Haji Ali Afzali, Jonathan Karnon

**Affiliations:** 1 College of Medicine and Public Health, Flinders University, Adelaide, SA, Australia; 2 Australian Institute of Health Innovation, Macquarie University, Sydney, NSW, Australia; 3 Rehabilitation, Aged and Extended Care, Southern Adelaide Local Health Network, Adelaide, SA, Australia

**Keywords:** early HTA, development-focused HTA, service technologies, healthcare delivery, modelling

## Abstract

**Objectives:**

We draw from the Health Technology Assessment (HTA) literature to propose how hospitals and local health networks can prepare the key components of early economic evaluations to support the development and management of health service interventions.

**Methods:**

Using the case example of a proposed intervention for older people in the Emergency Department (ED), a conceptual logic model of a new health service intervention is articulated to inform the structuring and population of a decision-analytic model using observed data on the existing care comparator and structured elicitation exercise of initial stakeholder expectations of intervention effects.

**Results:**

The elicited patient pathway probabilities and lengths of stay quantities profile which of the existing types of patients are expected to avoid the ED and how this impacts the lengths of stay across the system. The exercise also quantifies the stakeholders’ uncertainty and disagreement, with qualitative insights into why. The elicitation exercise participants draw upon the rationale for how the intervention is expected to affect a change within the local context, as captured within the logic model, together with the descriptive analyses of the characteristics and utilization of their target population. Feedback indicates the methods are acceptably robust yet pragmatic enough for healthcare delivery settings.

**Conclusions:**

As proposed in this paper, HTA methods can be used to capture how key stakeholders initially expect a service intervention to affect a change within their local context. The example results can be used in a decision-analytic model to guide the development and management of an intervention.

## Introduction

Health economists are often recruited to infer the value of healthcare and service interventions through observation *ex post*, following their implementation. For *ex post* analyses, data is required to have been collected prospectively alongside controlled trials or available through routine collection practices. However, normative reasoning is used *ex ante* to inform decisions about the local design and development of potential service interventions prior to their implementation (1).

While early and iterative cycles of evaluation and decision-making are nothing new (2), the greater uptake of *ex ante* evaluations and Health Technology Assessment (HTA) methods for normative reasoning in local service delivery settings requires health economists and health technology analysts to *“… break their addiction to technology adoption*” (3) and instead use economic evaluation in *“pathway management*” (4).

For this, methods can be translated from “early health technology assessment” (eHTA) that are employed within the medical product industry. Such methods include decision-analytic modeling to conduct scenario analyses, real options analysis, and headroom and threshold analyses to guide research and development prior to approaching decision-makers for purchasing and budgeting of a distinct or finalized technology (5). Others have referred to these methods as development-focused health technology assessment (DF-HTA), with Bouttell and Briggs (6) arguing that there is a fundamental difference in the focus rather than merely an early vs late temporal sequence. The key focus is informing a conceptualization of the conditions under which possible and expected cost-effectiveness may be achieved and profiling the extent of uncertainty and further evidence requirements. The distinction between evaluations for the purposes of exploring possible costs and outcomes rather than expected cost-effectiveness is often likened to being *formative* guidance rather than *summative* judgments (7).

Recent efforts have been undertaken to encourage the use of eHTA and DF-HTA methods within hospital and healthcare delivery settings as part of Hospital Based Health Technology Assessment (HB-HTA) (8) and “embedded economist” programs (9). However, as highlighted in the reviews by Stome, Moger (10) and Tummers, Kvaerner (11), service settings represent a new and challenging application context of economic evaluation and HTA methods. There are few published examples of evaluations for organizational or service innovations, particularly those that are *ex ante*, that are not merely built around specific and relatively interchangeable medical devices.

Specific method developments that can support formative guidance for services include those of the INTEGRATE-HTA project (12), which encourage analysts and assessment committees to explicitly conceptualize technologies within a broader systems context. Another helpful development has been the recently published reference protocol on the use of structured expert elicitation for HTA by Bojke and Soares (13), which details methodological options for dealing with missing data and calls for further applied studies to demonstrate and strengthen their use.

The aim of this paper is to propose how contemporary HTA methods may be used to prepare early economic evaluations that inform the development and management of service interventions. We do this through a case example from a Local Health Network (LHN) in Australia in which we have been embedded as academic health economists and researchers.

## Case example

As one component of their broader acute care services, the Southern Adelaide Health Network (SALHN) and South Australian Ambulance Service (SAAS) deliver services for acute, but non-emergency, management for older people. For this, they receive a mix of activity-based and block funding from state and Commonwealth governments, together with private insurance funding and patient out-of-pocket payments.

Older people often have complex medical, social, and rehabilitation needs and are high users of hospital emergency departments (ED) in Australia. The perspectives of health professionals are that the hospital ED is not suitable for older patients with low acuity problems because the time and physical space constraints hamper the conduct of comprehensive assessments and transitional care planning (14). A new service intervention under development by SALHN and the SAAS to address the needs of these older people, called the Complex And RestorativeE (CARE) service, involves multiple workforce, built-environment, information technology, and other consumables components spread across hospital, virtual, and home-based settings. Phone calls to the SAAS are triaged, and older people with low acuity conditions are diverted from the ED to another purposefully built site or receive a home visit from an community outreach team (15).

The teams developing and implementing the CARE service intervention do not routinely operate within an environment of controlled trials but are required to present business cases to the LHN executive and funding bodies to inform their decision-making on whether and to what extent to vary previously commissioned spending.

The project described herein was reviewed by the Southern Adelaide Human Research Ethics Committee and registered on the SALHN Quality Register (Reference number: 2198).

## Methods and analysis

To prepare for an early economic evaluation within the local SALHN and SAAS context, three complimentary methods were undertaken:

1. Conceptual modeling of the CARE service, to articulate the causal logic behind the intervention to inform the structure of a decision-analytic model,
2. Analyses of existing care for the target population using health systems data to profile the alternative against which the intervention could be compared, and
3. Elicitation of initial expectations of intervention effects, to be used in decision-analytic modeling and iteratively updated as further evidence emerges.

Articulated within the conceptual logic model is the target population; a description of components of the interventions and their in-situ comparators; and how these are causally linked to operational and health outputs. This “logic model,” as it is popularly referred to within healthcare delivery settings, articulates what in HTA is known as the Population, Intervention, Comparator, and Outcomes (PICO).

To profile the incremental costs and effects, the maintenance of existing care was chosen as the best comparator. Five datasets were linked together by SALHN using anonymized patient identifiers, spanning the ED, admitted hospital care, and patient mortality. Analyses included descriptive statistics of patient characteristics, pathway probabilities, lengths of stay (LOS), readmissions, and mortality.

The conceptual logic model and information on existing care were then presented to participants in the elicitation exercise to inform their expectations of possible intervention effects. We elicited their expectations in the form of quantitative estimates using both the “chips and bins” fixed-interval method and a lowest/highest credible intervals approach.

Each of these methods is described further below.

### Conceptual modelling

Guidance on logic modeling questions and the PICO template were based on the INTEGRATE-HTA Model by Wahlster and Brereton (12), including the potential impact of exogenous factors within the broader organization or health system that may influence the causal logic. Their PICO questions were supplemented with additional questions used previously by Hardwick and Pearson (16), to explicate enabling or dampening effects from implementation of the intervention. Further probing questions were informed by document analysis of project materials produced within the health system.

Potential staff and stakeholder interview participants were recruited through a purposive, peer-referral sampling approach facilitated by oversight committees of the LHN. Those identified as potential participants were invited via email for an interview by the research team and provided informed, written consent.

Presented in Supplementary Appendix 1 is the full set of interview questions on a template “canvas”. Feedback from participants was captured on a virtual “white-board” on which the template canvas had been loaded, with interviews conducted via videoconferencing. The outputs from these interviews were then transcribed and translated into diagrams in MS Visio for validation by the broader team.

### Analyses of existing care

Routinely collected hospital and health service data from the first 6 months of 2020 was used to profile existing care for the older adult population in the local SALHN setting. This observation period was unaffected by COVID-19, as it preceded the brief lockdowns that first occurred in South Australia in November 2020 and prior to the 2021 arrival of community transmission.

The linked data were analyzed within Stata and MS Excel to look at the proportion of the population who traversed different pathways and the associated resource utilization.

### Elicitation of initial expectations

The elicitation exercise was broken into five parts, namely (i) definition of aims; (ii) selection of the experts; (iii) design of the exercise; (iv) conduct of the exercise; and (v) feedback of the results and update where necessary. Our approach was based on Soares, Bojke (17) and the recent guidance for elicitation in HTA by Bojke, Soares (13).

#### Definition of the aims

The goal of elicitation is to obtain a robust, quantitative interpretation of the best available evidence. Our aim was therefore to build upon our understanding of existing care and quantify the expected patient pathways and outcomes resulting from the new CARE service intervention.

To populate a future decision-analytic model, the parameters of interest were identified as the pathway probabilities of patient progression through the health service and cumulated quantities along these pathways, including ED and inpatient length of stay (LOS).

#### Selection of the experts

A purposive, peer-referral sampling approach was taken. The sample included a diverse group of clinical and managerial stakeholders involved in the design and delivery of the intervention.

#### Design of the exercise

Visual aids were produced, based on Grigore and Peters (18). Included within Supplementary Appendix 2 is the full list of elicitation questions within the exercise that relate to the pathway probabilities. An example of the histogram for elicited proportions and calculations used for the descriptive statistics, is available in Supplementary Appendix 3.

For quantities associated with patient pathways (e.g., length of stay), participants were asked to provide their best estimate of mean effect (i.e., an Average Length of Stay, ALOS) together with their view on the lowest and highest credible intervals of possible mean effect. Supplementary Appendix 4 contains the full list of elicitation questions posed within the exercise that relate to the LOS quantities.

The distributions of probabilities and credible intervals of mean effects were elicited to profile second-order or epistemic uncertainty that represented the participants’ judgement of the imperfect knowledge of the true population effects.

#### Conduct of the exercise

The exercise was conducted as a 1-on-1 online interview via videoconferencing, following an online group seminar that introduced the concepts of elicitation, the quantities being elicited, and the method of elicitation. Participants were also provided with an introduction to cognitive biases, such as anchoring, availability, and overconfidence, which were then refreshed at the beginning of the exercise. Bias mitigation was a feature of the exercise, where participants were asked about the plausibility that their judgment was flawed and whether their range of possible values were valid. Throughout the exercise, respondents were encouraged to “think aloud” on their interpretation of the available evidence. A warm-up exercise using the visual aids was included.

#### Feedback and updating of the results

Box-and-whisker plots representing participants’ individual and pooled ranges of expectations were anonymized and fed back via email. This included a summary of the considerations/logic expressed by others. The considerations/logic were derived from “think aloud” transcripts that were thematically coded using QRS NVivo software. To capture their considered and reflective beliefs, each participant’s own scores were identified to them so that they could compare theirs to the group, self-assess the performance of their previously elicited results, and potentially update their original estimates. Supplementary Appendix 5 comprises the information that was fed back to participants, with the template to revise their expectations.

The individual feedback from participants was linearly pooled using a simple unweighted arithmetic average. To obtain estimates of mean effect and enable probabilistic sensitivity analyses (PSA), we fitted parametric continuous densities to the pooled results. For the elicited lengths of stay, least squares were calculated for normal, gamma, and lognormal distributions and considered when selecting and fitting the best distribution. Beta distributions were selected for all elicited proportions.

## Case example results

### Conceptual modelling

**Figure 1.**
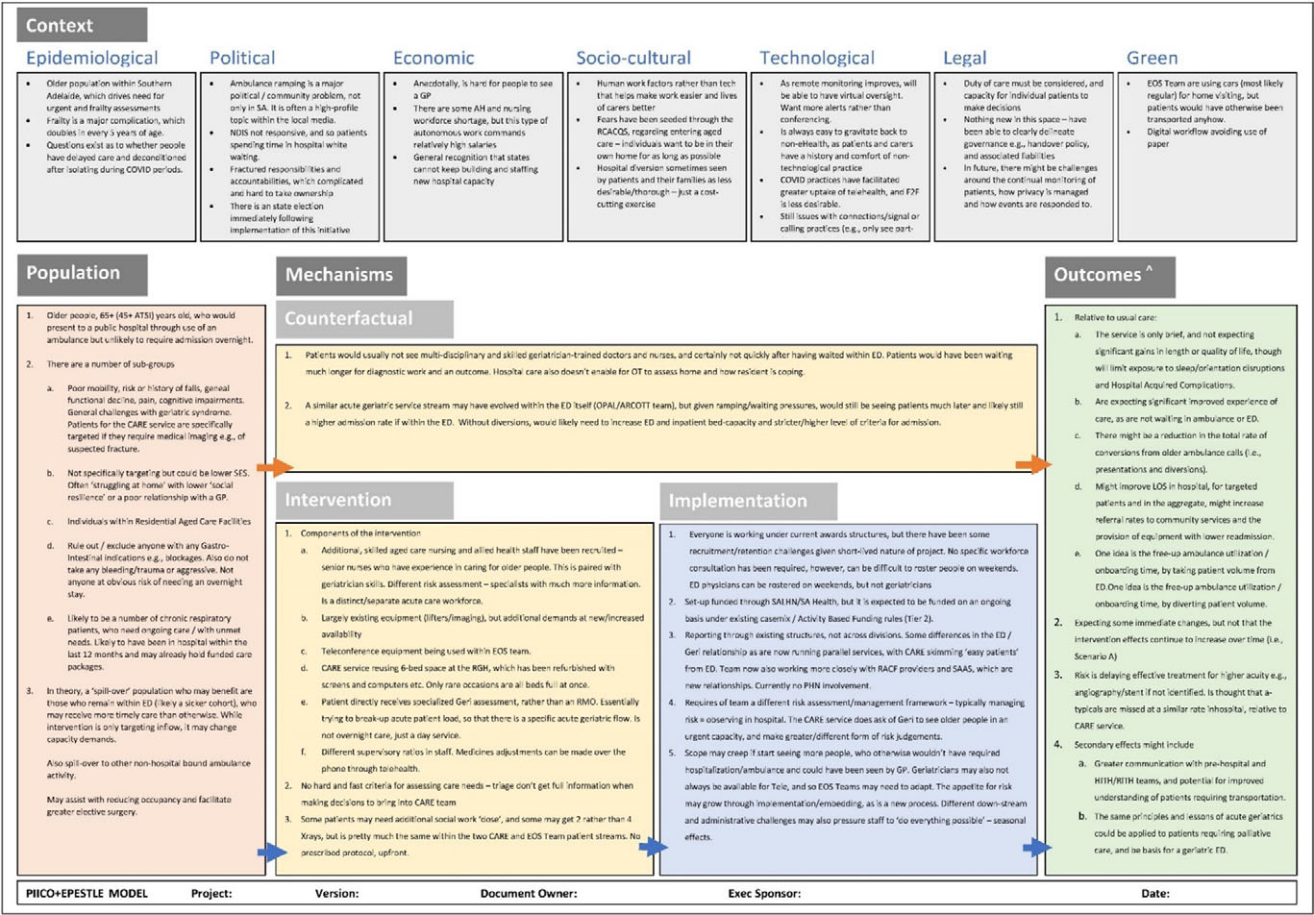
The synthesized feedback within the conceptual logic model.

The conceptual modeling for the CARE service intervention is detailed within Figure 1. It provides a synthesis of the logic behind how stakeholders expected the intervention to provide value in the local context.

Stakeholders said that the CARE service was being developed to target a broad population of older people aged 65+ who are thought to require urgent, but not acute, care. Those interviewed theorized several subgroups related to patient health conditions, socio-economic status, and living conditions. They also noted potential “spill-over populations” of indirect patient beneficiaries who would gain greater and sooner access to alleviated resources.

Additional skilled workforce were expected to be recruited, together with new teleconference equipment and the repurposing of a 6-bed space within a rehabilitation hospital setting. All patients identified as relevant for the CARE service were expected to receive largely the same “dose” of combined diagnostics, assessments, treatment, and discharge planning, with some variation in consumables and clinician hours (i.e., length of stay).

To implement the new model, existing workforce, funding, and governance policies and practices were expected to be sufficient. No specific consultation with employees on changes in role was envisaged, though it was noted that future iterations of the intervention may involve rostering staff on weekends and that this would require new policies and enterprise agreements. It was also acknowledged that there was the potential for professional tensions between physicians within the ED and geriatricians working within CARE. It was noted that the latter may be seen as “skimming the easy patients” from the ED and that any evaluation must address potential patient selection bias.

Relative to usual care, it was expected that there would not be any major impact on length or quality of life. There were, however, expectations that the new CARE service would provide superior patient experience and result in LOS reductions due to hospital avoidance. The effects of implementing the CARE service were expected to be immediate, and any minor increases over time from learning effects and improvement in implementation may be counteracted by a loss of operational focus on the intervention. The main learning curve affects expected on intervention effects over time were with regards to the conduct of referral assessments and the managing of clinical risk by geriatricians over shorter and often virtual consultations than are currently the standard of care. The risk of harm due to delayed effective treatment for falsely identified patients was not thought to be higher than existing inhospital rates of “missed, atypical patients.”

**Figure 2.**
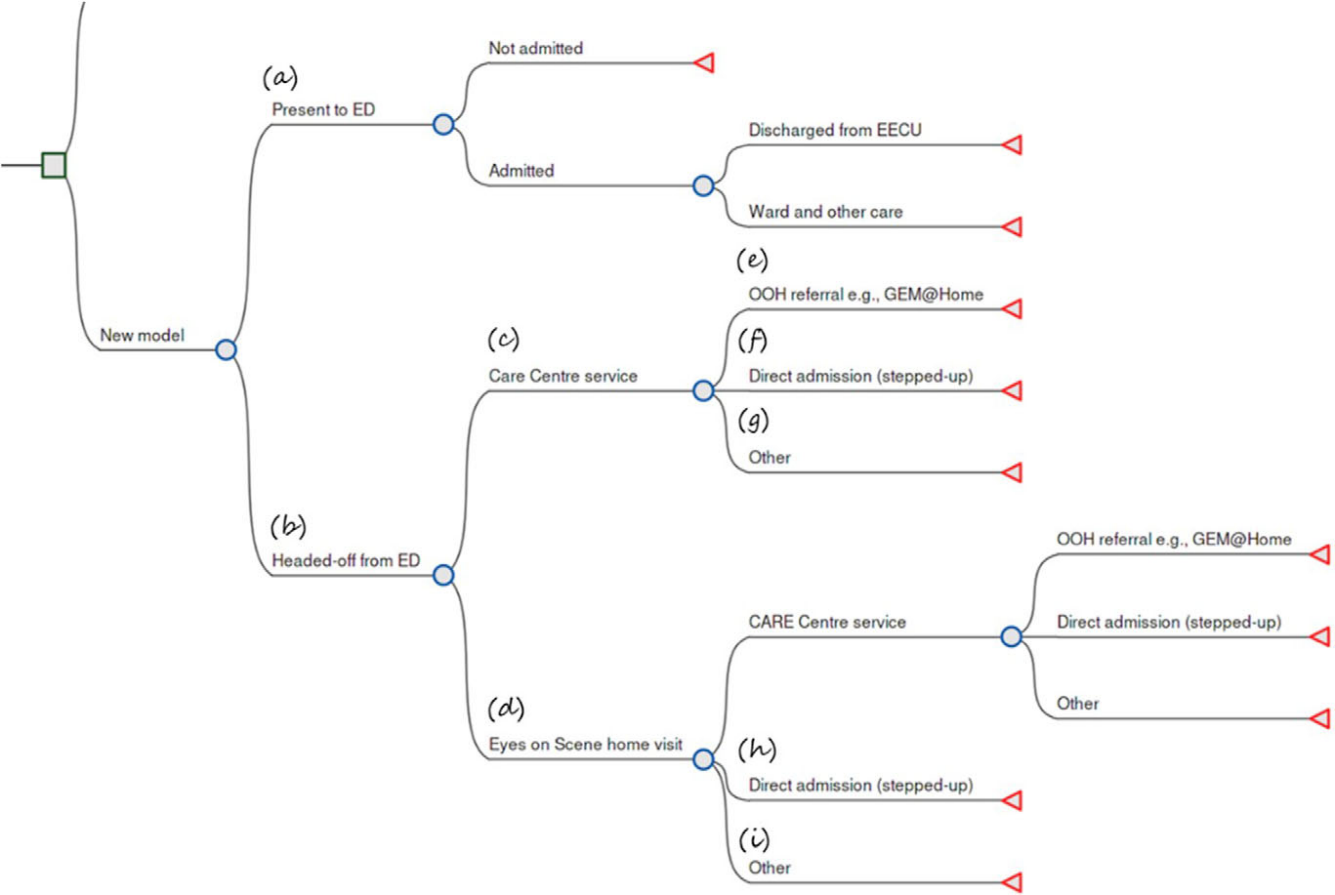
Structure of patient pathways with CARE service intervention.

Informed by the conceptual modeling, the expected CARE pathways were visualized as a decision tree in Figure 2, below. Under the CARE service intervention, there are a few different pathways related to either (a) continuing to present to the ED or (b) being headed off from the ED by either (c) attending a centrally located CARE Center, or (d) receiving a home visit from an Eyes on Scene team.

### Analyses of existing care

At a high level, existing care was profiled for a target population defined within the data as those who present to the ED via ambulance, aged 65 years or older, who receive an Australasian Triage Categorization of 3 to 5 (lower acuity), before then either being admitted or discharged from the ED. Those admitted were either discharged from the Extended Emergency Care Unit (ECCU) or taken into the hospital to receive ward and other care.

**Figure 3.**
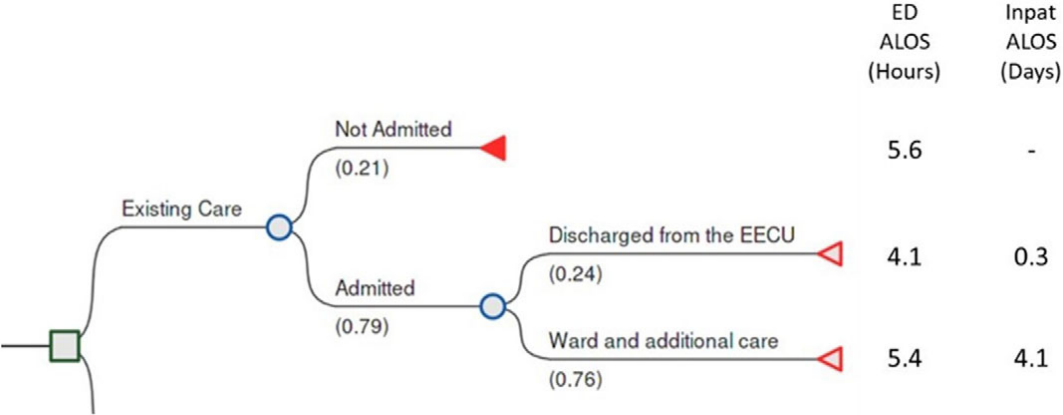
Existing care model populated with local health service data.

In the first half of 2020, it was found that there were 5,414 presentations to the ED by the target population. This represents an annualized figure of 10,828 or 210 per week or 30 per day. It was observed that 21 percent of these were not admitted and discharged from the ED with an ALOS of 5.6 hours. Of those 79 percent who were admitted, 24 percent were discharged from the Extended Emergency Care Unit (EECU) after an ALOS of 4.1 hours within the ED and 0.3 days (7.2 hours) in the admitted EECU space. The remaining 76 percent of those admitted were transitioned to care on an inpatient ward within the hospital, in which they stayed for an average of 4.1 days, following an average of 5.4 hours within the ED. These existing care figures are represented graphically within Figure 3, below. It is expected that the existing care represents a level and quality of activity within the LHN that would be sustained for this population group in the absence of the intervention and is therefore a suitable comparator to the CARE intervention.

### Elicitation of initial expectations

On average, the elicitation interviews took two hours to complete, with five participants provided the opportunity to complete the exercise across up to three sessions. One participant was lost to follow-up and did not complete the elicitation of expected lengths-stay effects, because of their change in role at the LHN and subsequent unavailability. The results of their elicited pathway probabilities were retained for analysis. One participant abstained from providing estimates of LOS within the CARE service. One participant elected to update their results following feedback on the initially elicited data. While both the original and updated scores were retained to be used in future “what if” scenario analyses, only the final updated scores are presented here within the results.

Presented in Table 1 are the pooled estimates from the elicitation exercise for the pathway probabilitie, and ALOS for ED, CARE Center and/or Eyes on Scene, and inpatient services. Full disaggregated results of the expert elicitation from individual participants, together with the pooled estimates for both pathway probabilities and length of stay, are provided in Supplementary Appendix 6.

**Table 1.**
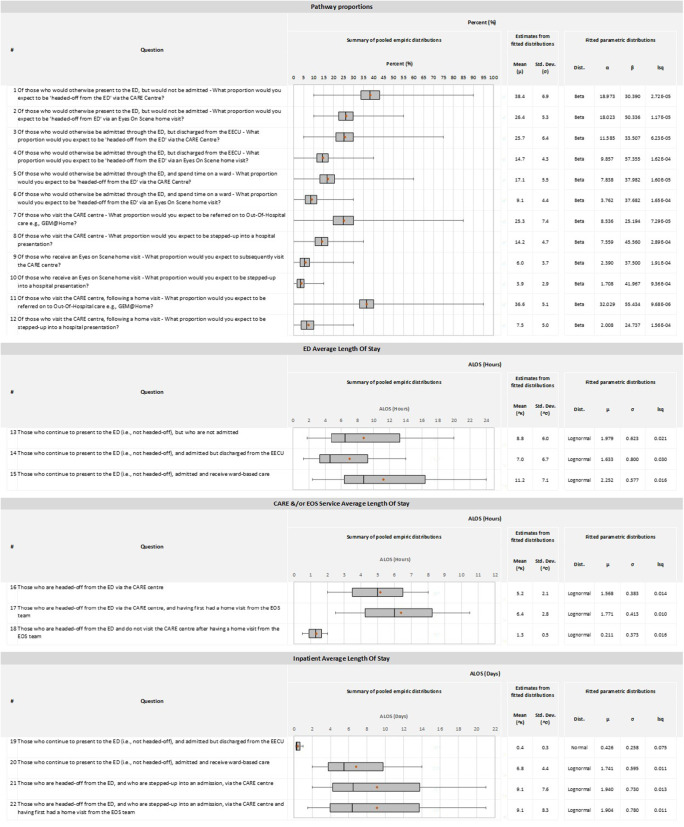
Pooled estimates of expected effects from the structured elicitation exercise

#### Pathway probabilities

The pooled pathway probabilities profile the early expectations of respondents for how the target population will be “headed-off from the ED” via the CARE Center and Eyes on Scene home visit. Of those who would otherwise be discharged home without an admission, be admitted through the ED but discharged from the EECU, or be admitted through the ED and spend time as an inpatient on the ward, the expected proportions of those headed off from the ED via the CARE Center are 38.4, 25.7 and 17.1 percent, respectively. For the same groups, the expected proportions of those headed off from the ED via an Eyes on Scene home visit are 38.4, 25.7, and 17.1 percent, respectively.

Once headed off, respondents indicated that they thought most of those who first visit the CARE Center will follow “other” pathways (38.5 percent), including going home, with 14.2 percent then expected to stepped-up into the hospital and be admitted, and the remaining 25.3 percent referred to an OOH service run by the LHN. By and large, those headed off via an Eyes on Scene home visit were expected to remain at home to receive care, with only 6 percent of home visits expected to be subsequently referred onto the CARE Center, and a smaller proportion of 3.9 percent to be stepped-up into the hospital.

The wide whiskers of the box plots indicate that respondents think there is significant uncertainty around several of the pathway probabilities. Individually, respondents were most uncertain about the proportions of ED patient groups who would be “headed-off” via the CARE Center, particularly for those non-admitted patients (Question 1).

The pooled results in Table 1 profile the extent of uncertainty expressed by the stakeholders; however, they do hide the inter-rater disagreement, which is evident within the individual responses to each question as shown in Supplementary Appendix 6.

**Figure 4.**
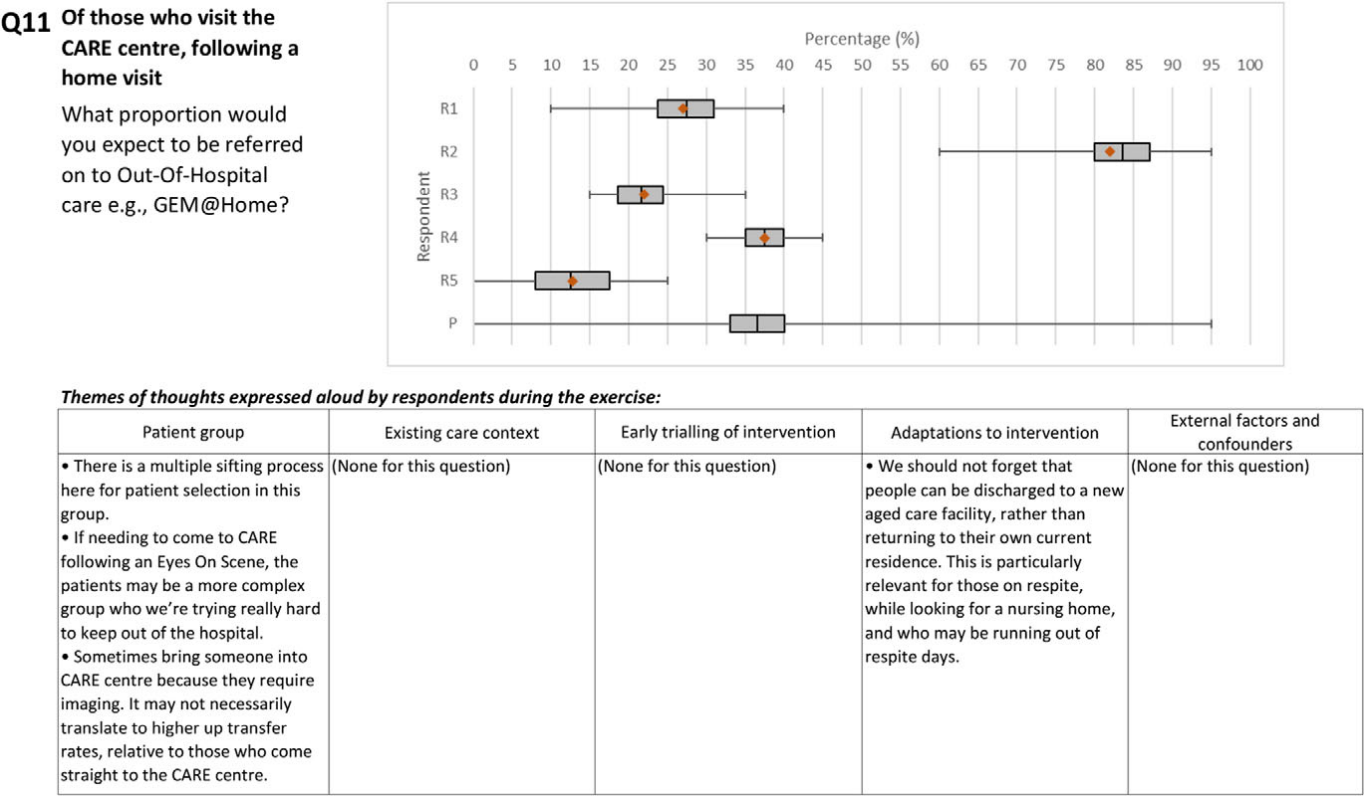
An illustration of individual responses underlying pooled estimates, using Question 11.

As an indicator of disagreement, the most variance in the elicited mean effect between respondents was elicited for Question 11. This question sought the expected proportion of patients referred onto further outof-Hospital care, following both a home visit and trip to the CARE center. However, as highlighted in Figure 4, the extent of the differences in expected mean effects for Question 11 was driven by the individual response from Respondent 2, who expressed a different view from the others, both quantitatively and qualitatively.

A complete account of the themes of thoughts expressed aloud by respondents during the elicitation exercise is available in Supplementary Appendix 5.

#### Lengths of stay or service

As shown in Table 1, in response to the questions about the ED ALOS for those who continue to present to the hospital, respondents expected it to range from 7.0 hours to 11.2 hours, depending on the patient discharge destination. Of those still admitted but discharged from the EECU, the expected ALOS was 0.4 days, or 9.6 hours. Patients in the target population still presenting and being admitted into the hospital for care on the ward were expected to be inpatients for an average of 6.8 days.

The ALOS for those in the CARE Center and receiving a homevisit by the Eyes on Scene team was expected to be 5.2 hours and 1.3 hours, respectively. While elicited in combination, the Eyes on Scene home visit and Care Center encounter was roughly the addition of those two quantities, at 6.4 hours. If stepped-up into the hospital from either the CARE Center or a Eyes on Scene home visit, the expectation was that the ALOS would be 9.1 days.

#### Feedback and face validity of elicitation exercise

None of the participants in the elicitation exercise had previously been involved in a study to formally capture expert opinion, though all of them stated that they had an at least “fair” level of statistical knowledge. Respondent 1 gave feedback that they found the exercise difficult to complete due to heterogeneity within the patient groups, while Respondent 2 said they found it extremely easy. The others provided middle scores. They all reported that the results presented to them faithfully captured their expectations of effects from the CARE intervention.

## Discussion

The methods proposed in this paper are examples of how health economists can prepare early economic evaluations to inform the development and management of service interventions. The results in the case example are inputs for subsequent decision-analytic modeling that underpin business case proposals, as part of an iterative formative assessment and ongoing funding decision-making process within the LHN. The explicit structuring of the decision-problem and causal logic behind how an intervention such as the CARE service is expected to provide value helps to ensure that subsequent quantitative analyses are informative. Moreover, it can help to “bring people on the journey” and foster a shared understanding amongst stakeholders of the intervention and its evaluation.

Unfortunately, qualitative studies within economic evaluation are rarely formalized or rigorously reported (19). This is to the detriment of the relevance and uptake of quantitative analyses, because the qualitative detailing of expected causal effects can assist in the interpretation of the validity and transferability of quantitative data (20).

The analysis of existing care shows it can help confirm the extent that there is an opportunity for improvement. Linking these analyses with the conceptual logic model and any published evidence on intervention effects is also important for grounding stakeholders’ early expectations in the local context. While there are challenges with capturing the diffuse effects and wider-system impacts of what are sometimes called “complex” interventions, the development of evidence for economic evaluation does not necessitate complex methods, so long as relevant inputs and outcomes of complex processes can be specified, measured, and valued (21).

That said, idiosyncratic and dynamic decision-contexts may require greater use of non-traditional methods such as elicitation. As is the context with many service developments, there were existed no previous analyses of a similar intervention trialed locally. Further, the published evidence only partially covered some of the components of the local CARE intervention and were trialed in contexts with different underlying resource capacities and capabilities. As such, there was no data from which to infer or directly transfer expected results, and a structured elicitation exercise was required to derive estimates of unknown intervention effects. When structured to mitigate cognitive biases, expert elicitation is a rigorous way to generate an early indication of whether the direction and scale of expected effects validate the qualitative conceptual logic (22).

As shown in the CARE service example, the exercise explicitly captures stakeholder uncertainty and enables the identification of where they disagree. While parametric distributions fitted to the elicited results enable PSA and Value of Information (VOI) assessments, the existing results already indicate potential areas requiring clarification and prospective data collection (e.g., population features and utilization in Q11). Running the elicitation as a “think aloud” exercise further helps stakeholders, analysts, and decision-makers probe experts’ causal reasoning and potentially update the logic model.

With service interventions like the CARE service, it can be difficult to attribute a dose response mechanism of effect to a specific intervention through a trial or observational study. This is because the costs and effectiveness of services can be heavily moderated by exogenous policy and operational shifts, including other interventions implemented adjacent to or on top of each other. While some inference of intervention costs and effects or estimation of parameters is sometimes possible, it is more likely that a further round of informed elicitation exercises will be required to interpret what is observed within the broader systems context. Stakeholders can continue to iteratively test and update their expectations and causal logic as and when further evidence becomes available.

The greatest challenge with employing these methods in healthcare delivery settings is that they need to fit into the dynamics of the local decision-making processes, which often involve rapid, collaborative decision-making. Being “embedded” and aware of service pressures does help to anticipate intervention development. The feedback from participants on the methods proposed in this paper indicates that they are acceptable in their current decision context and map to our experience that they are no more laborious than existing business case development.

While a less structured “finger in the wind” type elicitation could be pursued, as it is often done in healthcare delivery settings, it is important that whatever quantitative estimates used in decision analyses characterize the uncertainty of said estimates and capture reflective expectations. Decision-makers seem to have less appetite for uncertainty and a want of more robust HTA methods for business cases expected to have a significant budget impact. It also seems that where this want is not met with robust but pragmatic methods, the “quality improvement” projects that tend to arise outwardly ignore potential options that require additional resources. The opportunity to “finger-wick” options that may be of value often never arrives, perhaps in part due to their implicit uncertainty and susceptibility to contention. Similar to the aim of this paper, Gray and Thynne (23) have recently shown how robust yet pragmatic methods can be used to interpret published evidence alongside local data and context to help garner group support for additional spending to improve care within a healthcare delivery setting.

Building upon the proposed conceptual logic modeling and elicited estimates of effect, threshold or “headroom” analyses can now be conducted to test the maximum allowable service delivery costs, given expected revenues or budget that the expected effects would warrant. That said, opportunity costs and therefore threshold prices for outcomes are not well understood at the local level (24). Decision-analytic modeling based on the methods proposed in this paper will need to draw on various themes of stakeholder reasoning and elicited ranges of uncertainty to explore possible scenarios that may be considered cost-effective.

## Conclusions

As proposed in this paper, HTA methods such as conceptual modeling, existing care analyses, and structured elicitation can be used to capture how key stakeholders initially expect a service intervention to affect a change within their local context. The example results can be used in decision-analytic modeling to guide the development and management of the intervention.

## Supporting information

Partington et al. supplementary material Partington et al. supplementary material

## Supplementary material

The supplementary material for this article can be found at http://doi.org/10.1017/S0266462324000539.
