# Supplementary material for: Preparing early economic evaluations for the development and management of health service interventions
Source: Int J Technol Assess Health Care. 2024 Oct 18;40(1):e47. doi: 10.1017/S0266462324000539 (PMC11563182; doi:10.1017/S0266462324000539)
Supplement: Partington et al. supplementary material [file S0266462324000539sup001.zip › Appendix 1 Conceptual logic model questions.pdf]

Context

Have there been, or do you expect there to be, changes within the broader environment or context that is external to the interventions, but which might advance or impede it?

Epidemiological

e.g., growth in underlying health need, or a spike in incidence.

Political

e.g., expecting specific stimulus funding tied to health infrastructure grants, or competing/cross-purpose programmes with similar aims

Economic

e.g., unavailability of specialised workforce, except where it can be 'flown-in' from interstate

Socio-cultural

e.g., social expectation of consumer powers and autonomy

Technological

e.g., expected investments in electronic medical records, to come online soon

Legal

e.g., assisted dying and advance care directives, or clinician rights to private practice

Green

e.g., focus on minimising single-use consumables or carbon emissions

Population

1. Who is the patient or consumer population targeted by this intervention? [1]
2. Who are the possible sub-groups within this population that affect their health needs, ability to access services, and their ability to respond to treatment?
    a. Based on a person's individual characteristics and behaviors [2]
    b. Based on a person's social and economic environment [3]
    c. Based on a person's physical environment [4]
    d. Based on their response to treatment
    e. Based on the availability of existing service alternatives or enablers
3. Who are the other 'spill-over' populations, not being targeted by the intervention, but who may benefit? [5]
4. Who are any other 'participants' that are being targeted? [6]

Mechanisms

Counterfactual

1. Thinking through the similar components of the intervention – what do you think are the main differences between the intervention and usual care?
2. How would this service otherwise have been expected to evolve and change over-time?

Intervention

1. Can you walk me through the general process of the intervention, from start to end?
2. What do you think of as being the main components of the intervention?
    a. New or additional workforce capabilities or capacity?
    b. New or additional equipment and consumables being used?
    c. New or additional IT and communication systems?
    d. New or additional use of physical space?
    e. A change in the timing and sequence of care?
    f. A change in how it is being coordinated?
3. How is the intervention applied consistently and cumulatively (i.e., according to need)? [7]
4. Can you explain any different 'doses' of the intervention (i.e., the intensity and mix of components), tailored to different sub-groups?

Implementation

1. Have there been, or do you expect there to be, new policies to be required to implement the intervention? [8]
2. Have there been, or do you expect there to be, new funding mechanisms required to implement the intervention? [9]
3. Have there been, or do you expect there to be, changes to the structure of organisations involved in implementing the intervention? [10]
4. Have there been, or do you expect there to be, changes required of individuals who provide of care? [11]
5. How is the intervention expected to 'bend' or evolve during implementation and actual use?

Outcomes ^

1. Relative to usual care, what are the additional or new effects or outcomes that the intervention seeks to achieve for the target population?
    a. Gains in the length of life [12]
    b. Gains in health capabilities and quality of life[13]
    c. Greater experience of care [14]
    d. Process or surrogate outcomes [15]
    e. Minimisation of utilisation or costs [16 ^]
        - If minimization only - how are equivalency of other outcomes being guaranteed?
        - How will freed-up inputs/costs be captured for being re-purposed/re-invested or extracted?
2. How are any expected learning-effects or system adaptations likely to have an impact on the above effects, over time? [17]
3. How are any of these outcomes potentially negatively impacted by the intervention?
4. How do you think these primary effects drive any secondary effects?
    a. Down-stream outcomes for targeted or 'spill-over' patients
    b. Economies of scope / strengthened system

PIICO+EPESTLE TEMPLATE **Project:** **Version:** **Document Owner:** **Exec Sponsor:** **Date:**

1 e.g., a cohort who are typical and representative of the individuals who receive care

2 e.g., health-specific conditions, such as comorbidities; and person-specific traits such as gender, ethnicity, or personality

3 e.g., income, education, social isolation, employment, housing

4 e.g., access to and utilisation of services, living and working conditions

5 e.g., those receiving care adjacent to a target population in the same ward, or those who may alternatively be able to access 'freed-up' service capacity

6 e.g., informal carers

7 e.g., selection/exclusion criteria; guidelines; standardized coordination.

8 e.g., new organisational KPIs, or workforce rights to private practice.

9 e.g., greater use of NSW Health funding, or PHN funding, or Medicare funding, or private/self-funding or charity funding.

10 e.g., integration/coordination, cultural norms and expectations, expanded or reduced public/private/voluntary sector involvement.

11 e.g., roles and responsibilities of professional and informal carers, how they communicate, their motivations/desires/incentives, their experience.

12 e.g., higher survival rate or longer length of life

13 e.g., mobility, self-care, usual activities, pain/discomfort, and anxiety/depression

14 e.g., given sufficient and correct information, ease of communication, engagement in decision-making, personalised treatment, confidence in the service, perception of being well-organised, time spent waiting, overall satisfaction

15 e.g., able to maintain social network; maintain contact with GP; 'quality of care', hospitalisation rates, patient access, coping, patient self-efficacy/navigation, patient or carer engagement, staff experiences or outcomes

16 e.g., hospital length-of-stay, hospitalisation rates, use of equipment

17 see examples of time-series
