# Supplementary material for: Preparing early economic evaluations for the development and management of health service interventions
Source: Int J Technol Assess Health Care. 2024 Oct 18;40(1):e47. doi: 10.1017/S0266462324000539 (PMC11563182; doi:10.1017/S0266462324000539)
Supplement: Partington et al. supplementary material [file S0266462324000539sup001.zip › Appendix 2 SEE interview topic guide - Proportion.pdf]

Thanks for your participation

My name is [   ], and I am a part of the research team at Flinders University.

Thanks for agreeing to be interviewed.

Your participation is completely voluntary. Replying to our invitation email and acceptance of our invitation suggest written consent to take part in the study. If you change your mind, you can withdraw at any time and you do not have to give a reason. If you withdraw your consent, any information you may have already provided us will be deleted.

As mentioned in the information sheet, we would like you to give us your expert opinion on a few quantities, for which no empirical data is available. We will ask this information from you in several ways. You will be given a chance to adjust your answers and to provide feedback about the exercise.

We are also asking that, as much as possible, you 'think aloud' as we progress through the questions. We ask this because we're also wanting to understand your thought process and reasoning behind your responses.

Please feel free to ask me any question at any time during the interview. You may also contact the Flinders Health and Medical Research Institute (FHMRI) following the interview if you have any questions related to the study.

I will now turn on the tape-recorder (or Zoom/MS Teams recorder) and ask you a series of questions about the CARE service initiative. Please feel free to turn your video-off and follow along with the Mural whiteboard. Before we begin though, can you confirm whether you consent to being recorded?

[Start recording]

Expected ***<u>future</u>*** values, based on evidence, experience, and judgements

1. *Judgement by* ***anchoring***
… fixing your response to a question, based on a value provided earlier

2. *Judgement by* ***availability***
… attributing a higher probability to events that are familiar

3. *Being* ***overconfident***
… having a too narrow range of possibilities

Of those who would have historically presented to the ED and been discharged home …

Under the new model of care, what % or proportion would you expect over the next 12 months, to be 'headed-off from the ED' *<u>via the Care Centre</u>* ?

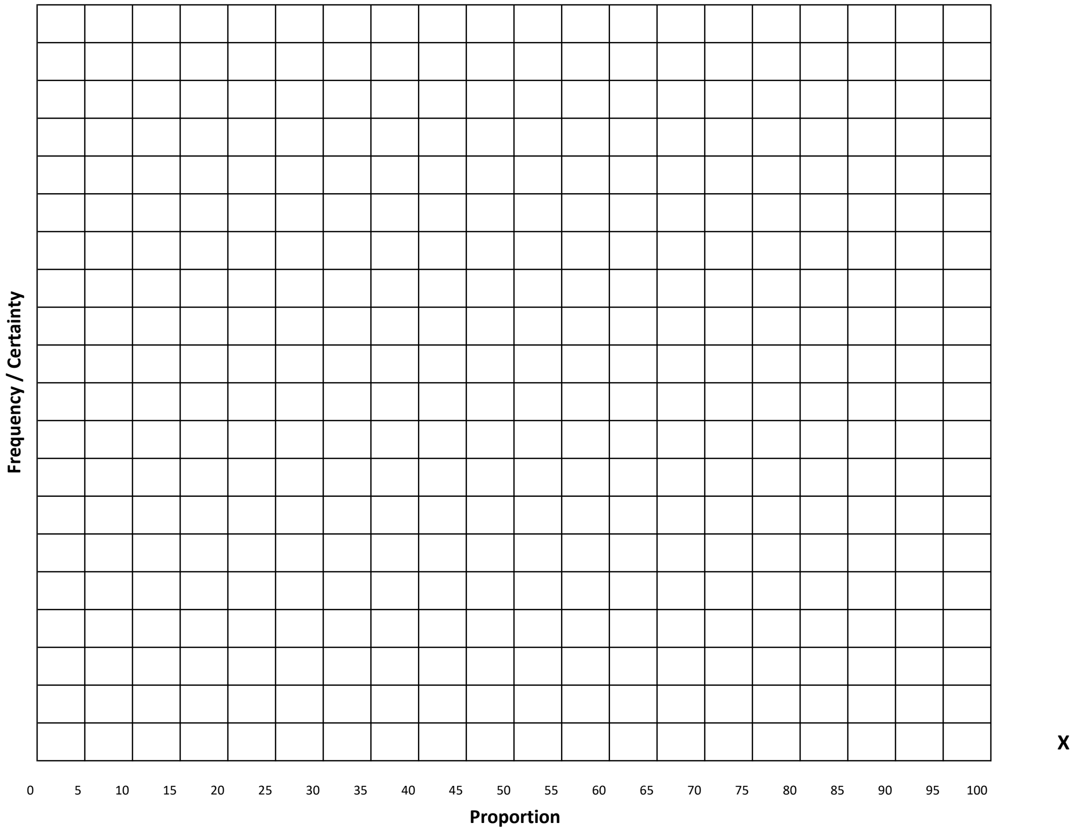

Each column represents a range of 5% of the proportion

Together, we'll place 20 x's in each grid

Each x = 5% likelihood that the *true* proportion value would be in the corresponding range

Starting from the bottom row, we can think about the full ***range*** of likely true values (e.g., proportion of patient who have a dog), and then stack crosses on top of this as necessary, to express your current belief of the ***strength*** of certainty of the quantity within that range.

Suppose that at some time in the future somebody tells you that the value of X has now been established quite accurately, and that its value is less/greater than your lowest/highest limit. What would be your reaction?

(a) Accept that this is plausible and admit that your judgement was flawed? Or

(b) Strongly suspect that the method used to determine this value was flawed or biased.

NOTES:

Can also think of it in terms of 'successes' within a sample size of 100

Of those who would have historically presented to the ED and been discharged home …

Under the new model of care, what % or proportion would you expect over the next 12 months, to be 'headed-off from the ED' _via an Eyes on Scene home visit_ ?

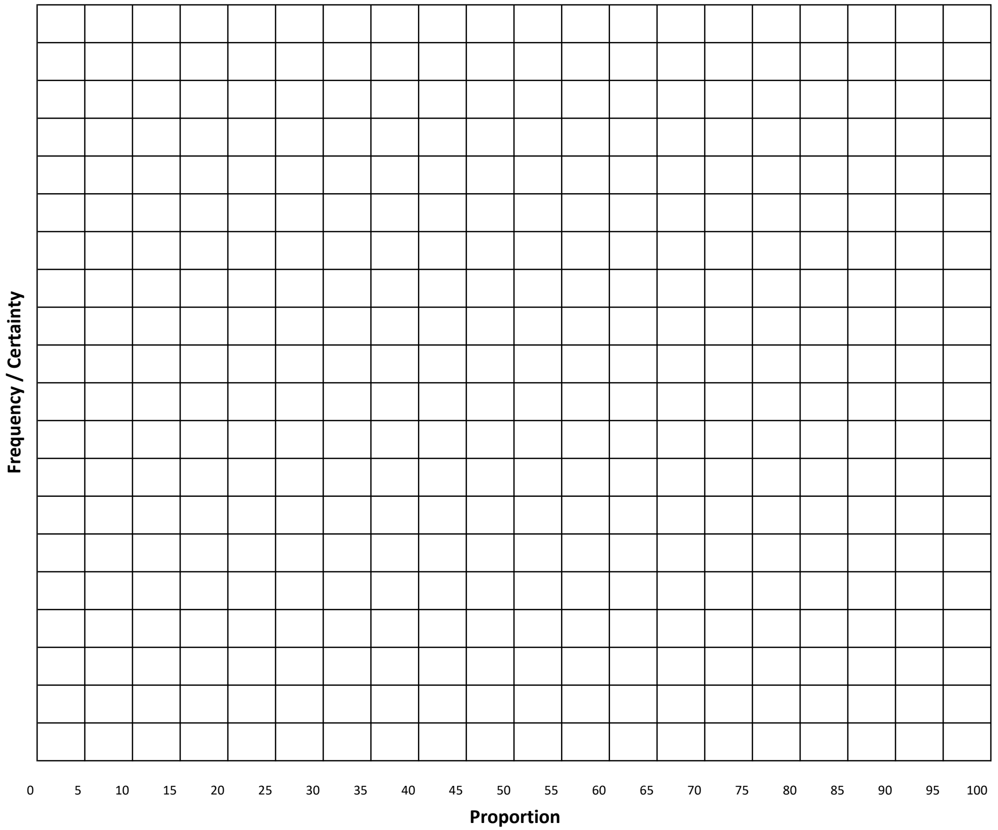

Each column represents a range of 5% of the proportion

Together, we'll place 20 x's in each grid

Each x = 5% likelihood that the *true* proportion value would be in the corresponding range

Starting from the bottom row, we can think about the full ***range*** of likely true values (e.g., proportion of patient who have a dog), and then stack crosses on top of this as necessary, to express your current belief of the ***strength*** of certainty of the quantity within that range.

X

Suppose that at some time in the future somebody tells you that the value of X has now been established quite accurately, and that its value is less/greater than your lowest/highest limit. What would be your reaction?

(a) Accept that this is plausible and admit that your judgement was flawed? Or

(b) Strongly suspect that the method used to determine this value was flawed or biased.

NOTES:

Check:

What is the sum total of those being diverted through the CARE and EOS pathways?

Does the total reflect your expectations, and not equal more than 100%?

Of those who would have historically presented to the ED, before being admitted but then discharged from the EECU …

Under the new model of care, what % or proportion would you expect over the next 12 months, to be 'headed-off from the ED' *via the Care Centre* ?

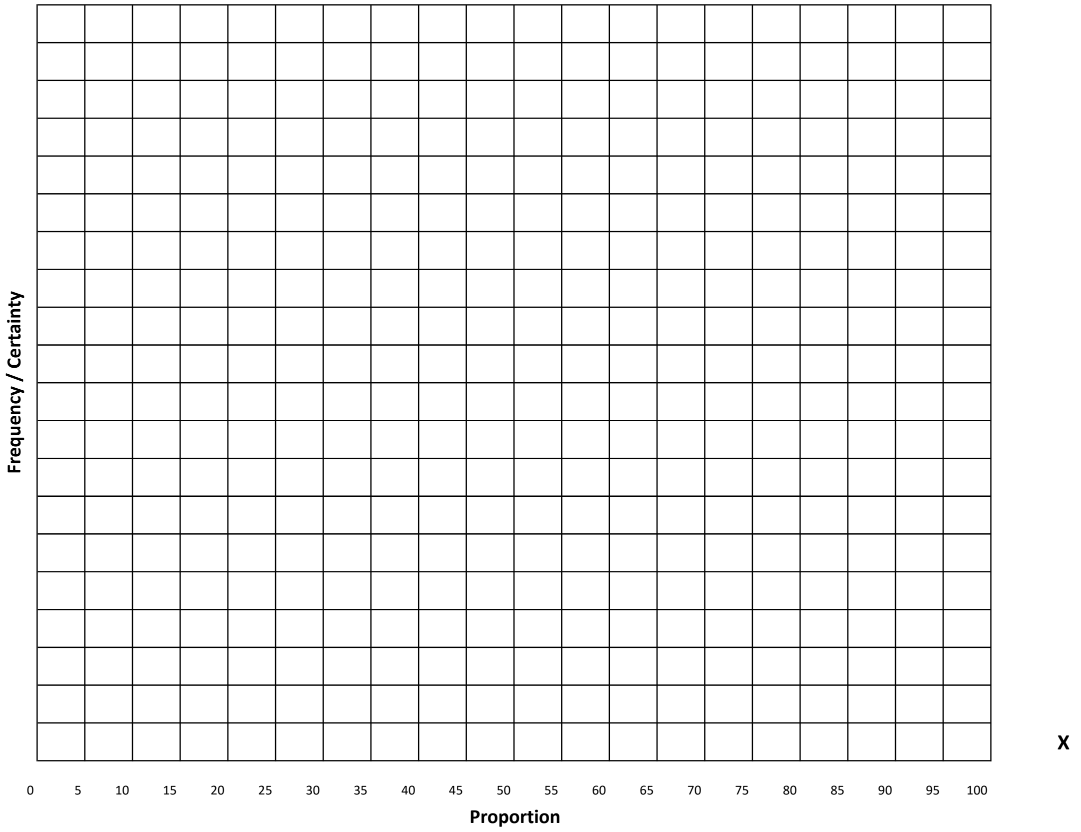

Each column represents a range of 5% of the proportion

Together, we'll place 20 x's in each grid

Each x = 5% likelihood that the *true* proportion value would be in the corresponding range

Starting from the bottom row, we can think about the full ***range*** of likely true values (e.g., proportion of patient who have a dog), and then stack crosses on top of this as necessary, to express your current belief of the ***strength*** of certainty of the quantity within that range.

Suppose that at some time in the future somebody tells you that the value of X has now been established quite accurately, and that its value is less/greater than your lowest/highest limit. What would be your reaction?

(a) Accept that this is plausible and admit that your judgement was flawed? Or

(b) Strongly suspect that the method used to determine this value was flawed or biased.

NOTES:

Of those who would have historically presented to the ED, before being admitted but then discharged from the EECU …

Under the new model of care, what % or proportion would you expect over the next 12 months, to be 'headed-off from the ED' *via an Eyes on Scene home visit* ?

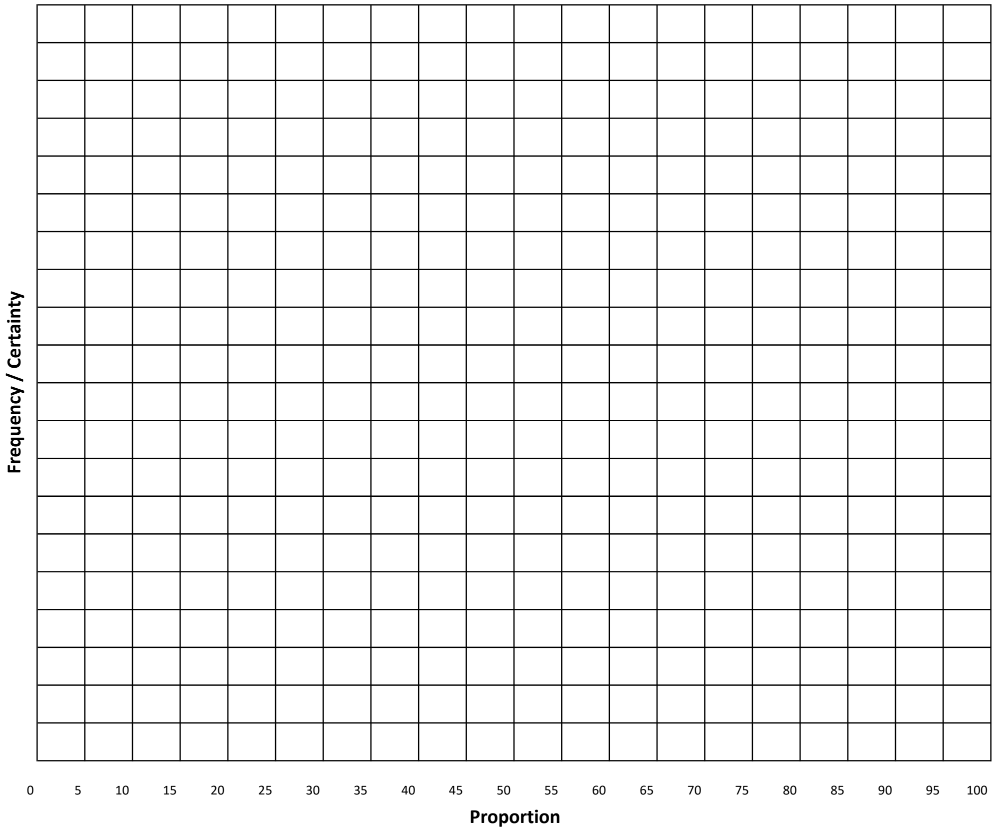

X

Each column represents a range of 5% of the proportion

Together, we'll place 20 x's in each grid

Each x = 5% likelihood that the *true* proportion value would be in the corresponding range

Starting from the bottom row, we can think about the full ***range*** of likely true values (e.g., proportion of patient who have a dog), and then stack crosses on top of this as necessary, to express your current belief of the ***strength*** of certainty of the quantity within that range.

Suppose that at some time in the future somebody tells you that the value of X has now been established quite accurately, and that its value is less/greater than your lowest/highest limit. What would be your reaction?

(a) Accept that this is plausible and admit that your judgement was flawed? Or

(b) Strongly suspect that the method used to determine this value was flawed or biased.

NOTES:

Check:

What is the sum total of those being diverted through the CARE and EOS pathways?

Does the total reflect your expectations, and not equal more than 100%?

Of those who would have historically presented to the ED, before being admitted and receiving care on the ward …

Under the new model of care, what % or proportion would you expect over the next 12 months, to be 'headed-off from the ED' <u>*via the Care Centre*</u> ?

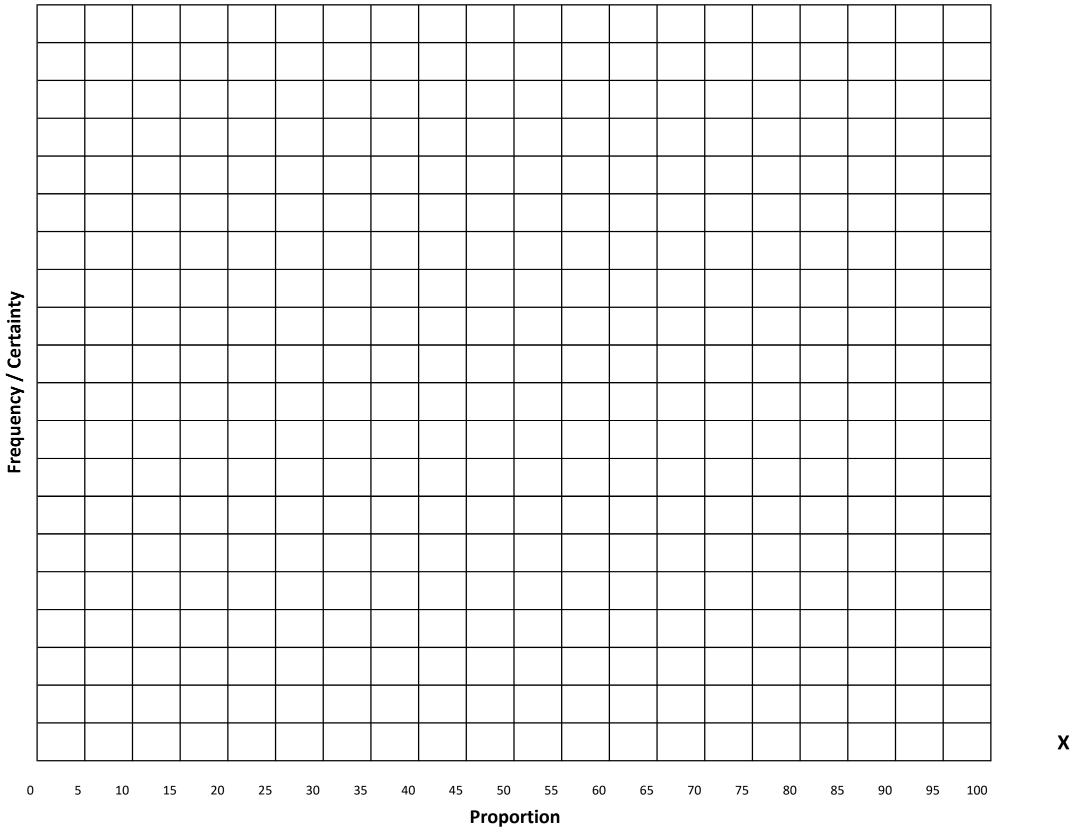

Each column represents a range of 5% of the proportion

Together, we'll place 20 x's in each grid

Each x = 5% likelihood that the *true* proportion value would be in the corresponding range

Starting from the bottom row, we can think about the full ***range*** of likely true values (e.g., proportion of patient who have a dog), and then stack crosses on top of this as necessary, to express your current belief of the ***strength*** of certainty of the quantity within that range.

Suppose that at some time in the future somebody tells you that the value of X has now been established quite accurately, and that its value is less/greater than your lowest/highest limit. What would be your reaction?

(a) Accept that this is plausible and admit that your judgement was flawed? Or

(b) Strongly suspect that the method used to determine this value was flawed or biased.

NOTES:

Of those who would have historically presented to the ED, before being admitted and receiving care on the ward …

Under the new model of care, what % or proportion would you expect over the next 12 months, to be 'headed-off from the ED' *via an Eyes on Scene home visit* ?

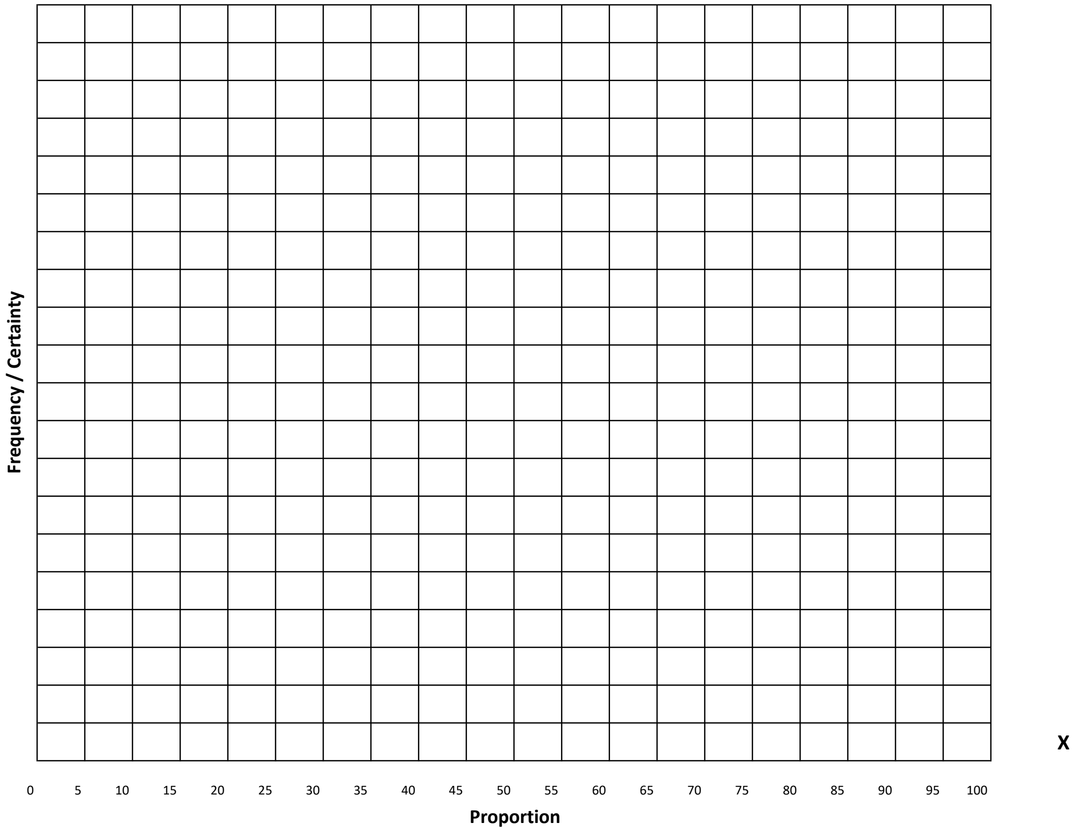

Each column represents a range of 5% of the proportion

Together, we'll place 20 x's in each grid

Each x = 5% likelihood that the *true* proportion value would be in the corresponding range

Starting from the bottom row, we can think about the full ***range*** of likely true values (e.g., proportion of patient who have a dog), and then stack crosses on top of this as necessary, to express your current belief of the ***strength*** of certainty of the quantity within that range.

Suppose that at some time in the future somebody tells you that the value of X has now been established quite accurately, and that its value is less/greater than your lowest/highest limit. What would be your reaction?

(a) Accept that this is plausible and admit that your judgement was flawed? Or

(b) Strongly suspect that the method used to determine this value was flawed or biased.

NOTES:

Check:

What is the sum total of those being diverted through the CARE and EOS pathways?

Does the total reflect your expectations, and not equal more than 100%?

Of those who visit the CARE centre

What % or proportion would you expect to be referred onto LHN-provided Out-Of-Hospital care e.g., GEM@Home?

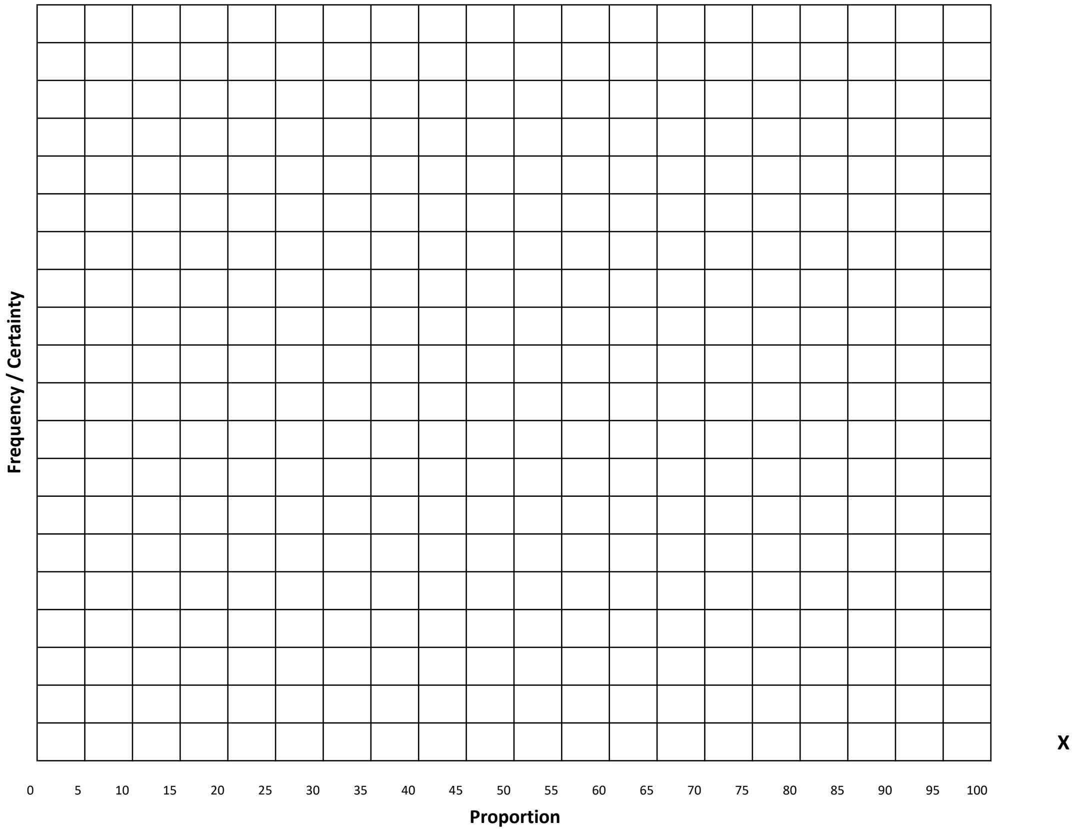

Each column represents a range of 5% of the proportion

Together, we'll place 20 x's in each grid

Each x = 5% likelihood that the *true* proportion value would be in the corresponding range

Starting from the bottom row, we can think about the full ***range*** of likely true values (e.g., proportion of patient who have a dog), and then stack crosses on top of this as necessary, to express your current belief of the ***strength*** of certainty of the quantity within that range.

Suppose that at some time in the future somebody tells you that the value of X has now been established quite accurately, and that its value is less/greater than your lowest/highest limit. What would be your reaction?

(a) Accept that this is plausible and admit that your judgement was flawed? Or

(b) Strongly suspect that the method used to determine this value was flawed or biased.

NOTES:

Of those who visit the CARE centre

What % or proportion would you expect to be stepped-up into a hospital presentation?

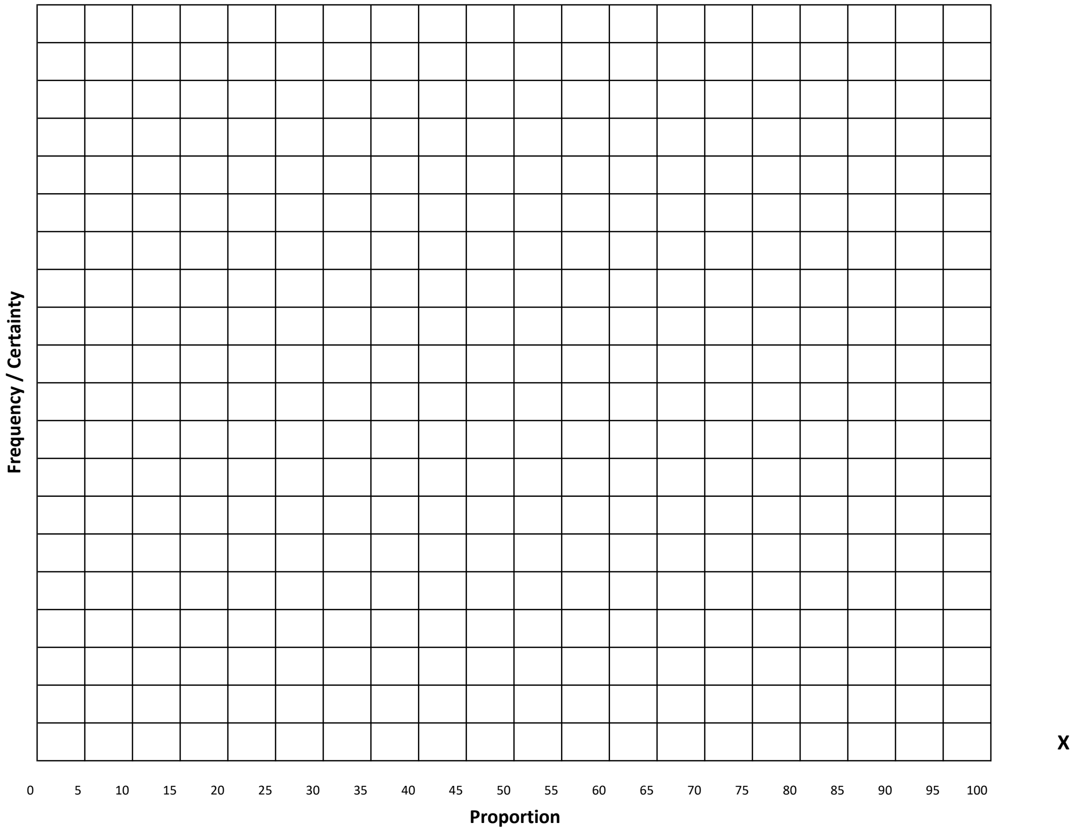

Each column represents a range of 5% of the proportion

Together, we'll place 20 x's in each grid

Each x = 5% likelihood that the *true* proportion value would be in the corresponding range

Starting from the bottom row, we can think about the full ***range*** of likely true values (e.g., proportion of patient who have a dog), and then stack crosses on top of this as necessary, to express your current belief of the ***strength*** of certainty of the quantity within that range.

Suppose that at some time in the future somebody tells you that the value of X has now been established quite accurately, and that its value is less/greater than your lowest/highest limit. What would be your reaction?

(a) Accept that this is plausible and admit that your judgement was flawed? Or

(b) Strongly suspect that the method used to determine this value was flawed or biased.

NOTES:

Of those who receive an Eyes on Scene home visit

What % or proportion would you expect to subsequently visit the CARE centre?

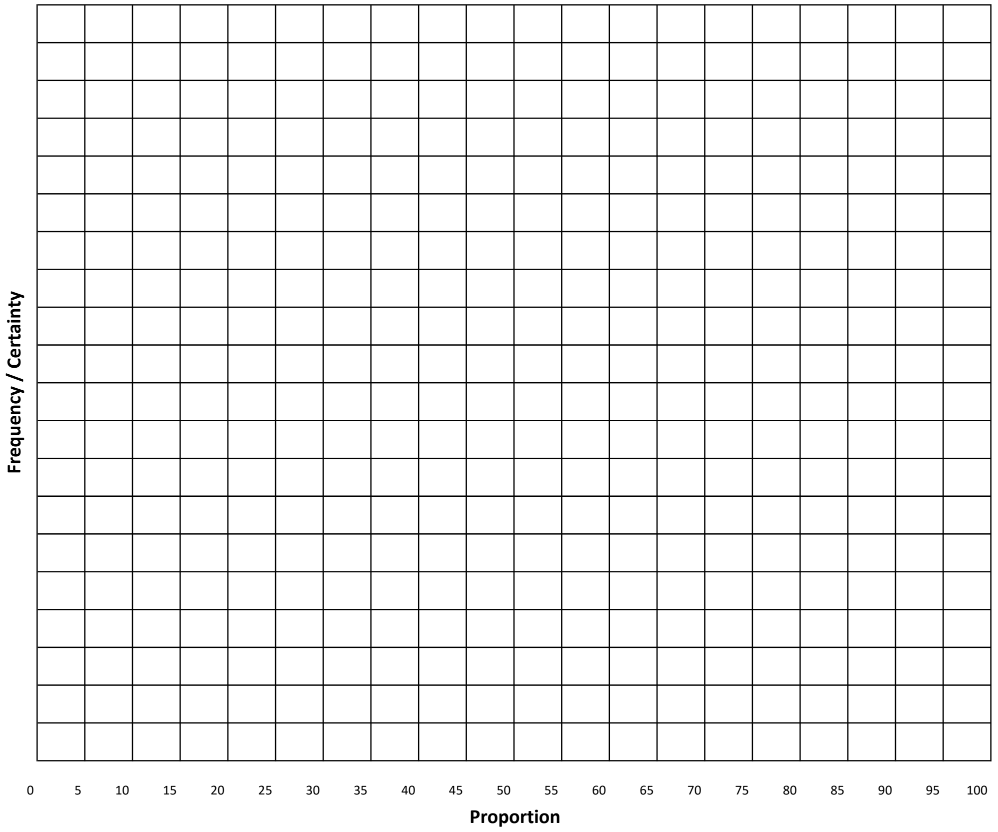

X

Each column represents a range of 5% of the proportion

Together, we'll place 20 x's in each grid

Each x = 5% likelihood that the *true* proportion value would be in the corresponding range

Starting from the bottom row, we can think about the full ***range*** of likely true values (e.g., proportion of patient who have a dog), and then stack crosses on top of this as necessary, to express your current belief of the ***strength*** of certainty of the quantity within that range.

Suppose that at some time in the future somebody tells you that the value of X has now been established quite accurately, and that its value is less/greater than your lowest/highest limit. What would be your reaction?

(a) Accept that this is plausible and admit that your judgement was flawed? Or

(b) Strongly suspect that the method used to determine this value was flawed or biased.

NOTES:

Of those who receive an Eyes on Scene home visit

What % or proportion would you expect to be stepped-up into a hospital presentation?

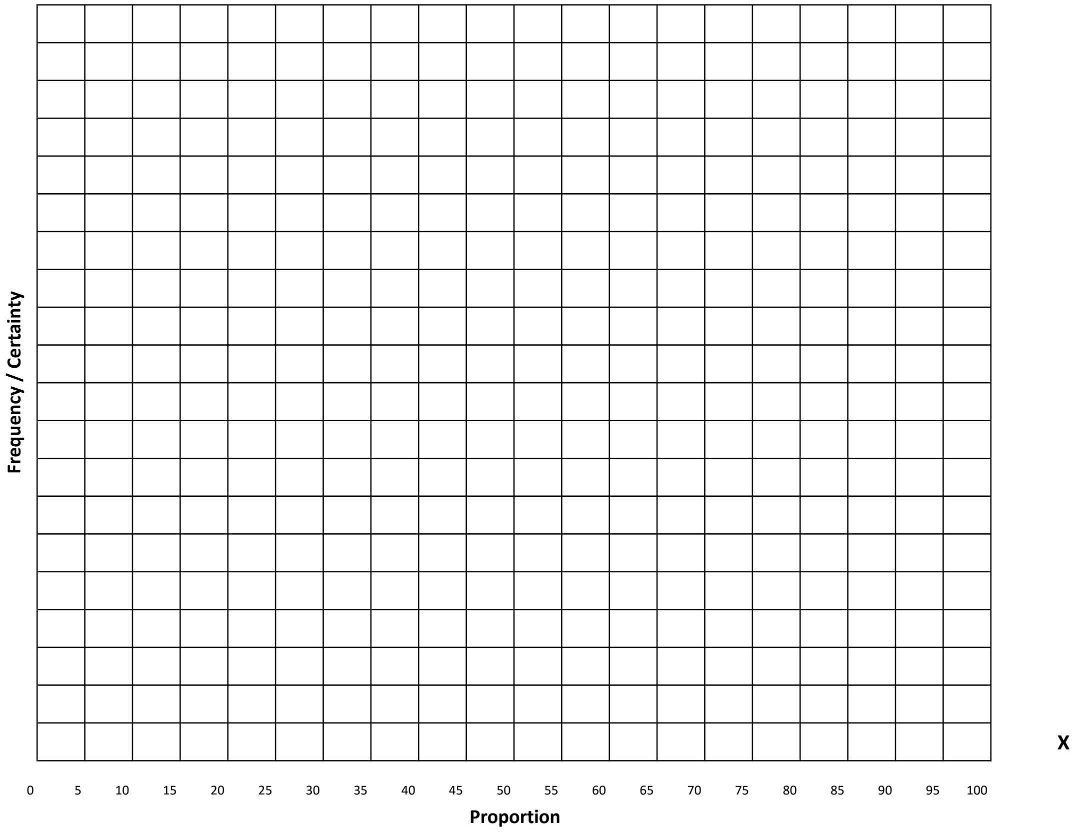

Each column represents a range of 5% of the proportion

Together, we'll place 20 x's in each grid

Each x = 5% likelihood that the *true* proportion value would be in the corresponding range

Starting from the bottom row, we can think about the full ***range*** of likely true values (e.g., proportion of patient who have a dog), and then stack crosses on top of this as necessary, to express your current belief of the ***strength*** of certainty of the quantity within that range.

Suppose that at some time in the future somebody tells you that the value of X has now been established quite accurately, and that its value is less/greater than your lowest/highest limit. What would be your reaction?

(a) Accept that this is plausible and admit that your judgement was flawed? Or

(b) Strongly suspect that the method used to determine this value was flawed or biased.

NOTES:

Of those who visit the CARE centre, following a home visit

Are the %s different from Answers to earlier questions in (D)?

NOTES:

Of those who visit the CARE centre, following a home visit

What % or proportion would you expect to be referred onto LHN-provided Out-Of-Hospital care e.g., GEM@Home?

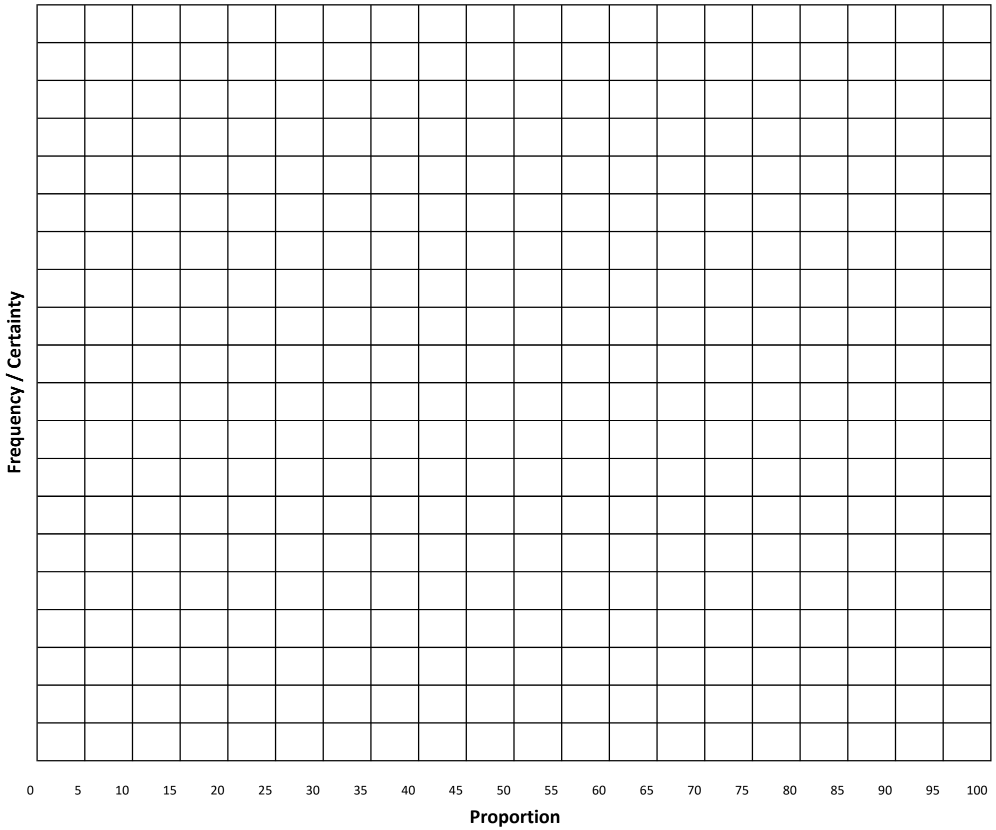

X

Each column represents a range of 5% of the proportion

Together, we'll place 20 x's in each grid

Each x = 5% likelihood that the *true* proportion value would be in the corresponding range

Starting from the bottom row, we can think about the full ***range*** of likely true values (e.g., proportion of patient who have a dog), and then stack crosses on top of this as necessary, to express your current belief of the ***strength*** of certainty of the quantity within that range.

Suppose that at some time in the future somebody tells you that the value of X has now been established quite accurately, and that its value is less/greater than your lowest/highest limit. What would be your reaction?

(a) Accept that this is plausible and admit that your judgement was flawed? Or

(b) Strongly suspect that the method used to determine this value was flawed or biased.

NOTES:

Of those who visit the CARE centre, following a home visit

What % or proportion would you expect to be stepped-up into a hospital presentation?

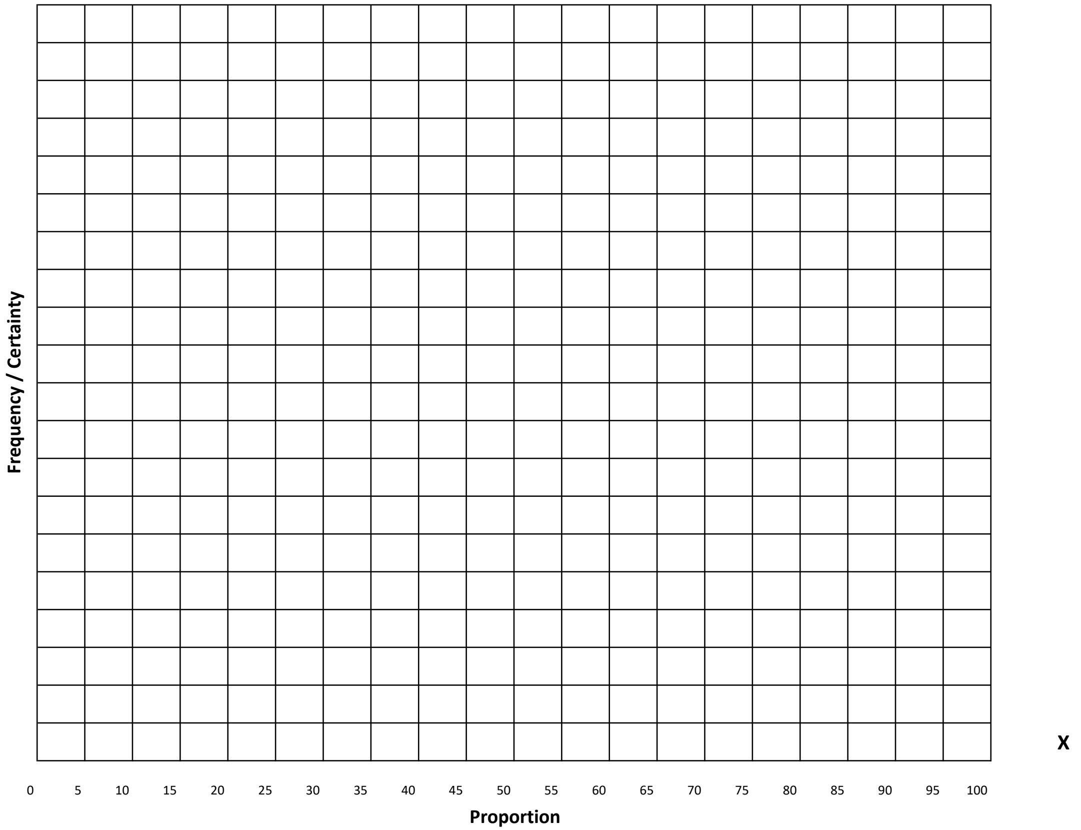

Each column represents a range of 5% of the proportion

Together, we'll place 20 x's in each grid

Each x = 5% likelihood that the *true* proportion value would be in the corresponding range

Starting from the bottom row, we can think about the full ***range*** of likely true values (e.g., proportion of patient who have a dog), and then stack crosses on top of this as necessary, to express your current belief of the ***strength*** of certainty of the quantity within that range.

Suppose that at some time in the future somebody tells you that the value of X has now been established quite accurately, and that its value is less/greater than your lowest/highest limit. What would be your reaction?

(a) Accept that this is plausible and admit that your judgement was flawed? Or

(b) Strongly suspect that the method used to determine this value was flawed or biased.

NOTES:

I would like to ask you a few questions about your experience with these types of statistical methods

C1. Have you participated before in exercises that formally capture expert opinion?

C2. What type of exercises?

C3. Would you consider your level of statistical knowledge to be:

Poor	Limited	Fair	Good	Excellent

C4. What potential conflicts of interest might you have that could be perceived as influencing your responses today?

F1.A Please consider how difficult it was for you to complete this exercise.

On a scale from 1 to 5, where 1 means "extremely difficult to complete" and 5 means "extremely easy to complete", how difficult did you consider this exerci

F1.B Please think about the difficulty of this exercise.

Can you indicate any specific obstacles you encountered in completing it?

F2.A Now consider how faithfully this exercise has captured your belief about the elicited value.

On a scale from 1 to 5, where 1 means "not at all faithful" and 5 means "exactly as I believe", how would you rate the face validity of this exercise?

F2.B Now please think about the "face validity" of this task (how successfully have we recorded your exact belief).

Can you indicate any specific obstacles that prevented the faithful capture of your belief?

F3.A Do you have any questions about the session we had today?

CLOSURE

Thank you very much for taking part in this study.
