# Supplementary material for: Preparing early economic evaluations for the development and management of health service interventions
Source: Int J Technol Assess Health Care. 2024 Oct 18;40(1):e47. doi: 10.1017/S0266462324000539 (PMC11563182; doi:10.1017/S0266462324000539)
Supplement: Partington et al. supplementary material [file S0266462324000539sup001.zip › Appendix 3 Calculations for histogram quantiles.pdf]

Of those who would otherwise present to the ED, but would not be admitted

What % or proportion would you expect to be 'headed-off from the ED' *via the Care Centre* ?

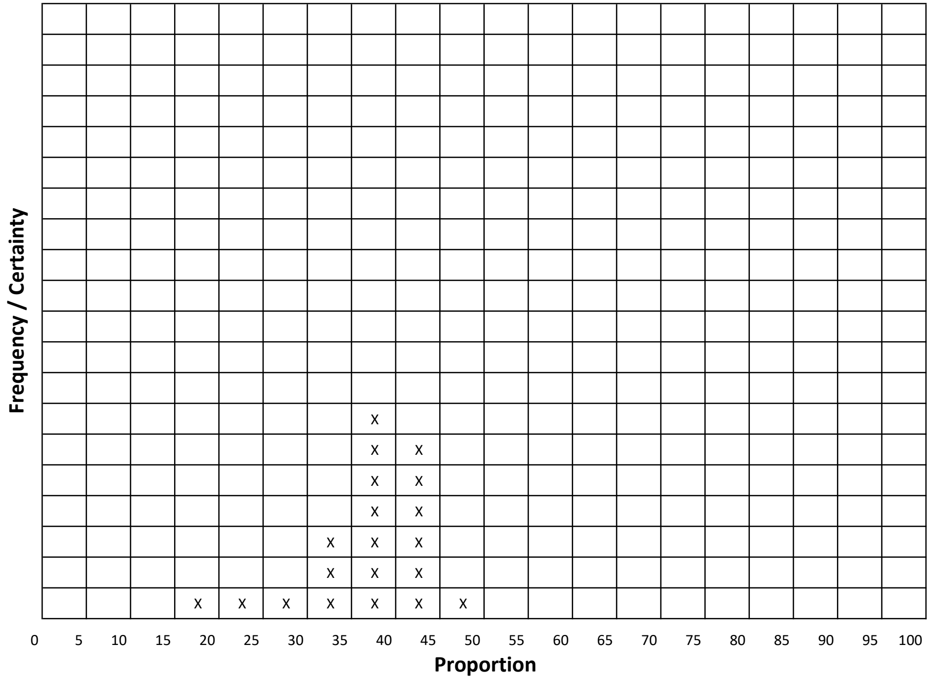

**m_i**	2.5	7.5	12.5	17.5	22.5	27.5	32.5	37.5	42.5	47.5	52.5	57.5	62.5	67.5	72.5	77.5	82.5	87.5	92.5	97.5
**n_i**	0.0	0.0	0.0	1.0	1.0	1.0	3.0	7.0	6.0	1.0	0.0	0.0	0.0	0.0	0.0	0.0	0.0	0.0	0.0	0.0
**Cumulative n_i**	0.0	0.0	0.0	1.0	2.0	3.0	6.0	13.0	19.0	20.0	20.0	20.0	20.0	20.0	20.0	20.0	20.0	20.0	20.0	20.0
**m_i*n_i**	0.0	0.0	0.0	17.5	22.5	27.5	97.5	262.5	255.0	47.5	0.0	0.0	0.0	0.0	0.0	0.0	0.0	0.0	0.0	0.0
**n_i*$(m_i - \mu)^2$**	0.0	0.0	0.0	361.0	196.0	81.0	48.0	7.0	216.0	121.0	0.0	0.0	0.0	0.0	0.0	0.0	0.0	0.0	0.0	0.0

**Min**	15	15
**Q1**	33	33.3
**Median**	38	37.9
**Q3**	42	41.7
**Max**	50	50

**Mean (μ)**	37
**Std. Dev. (σ)**	7

Estimate of mean (μ) = $\frac{\sum m_i n_i}{N}$

where:

m_i: The midpoint of the *i* th bin

n_i: The frequency of the *i* th bin

N: The total sample size

Estimate of median = $L + \left(\frac{N/_2 - F}{f}\right) \times w$

where:

L: The lower limit of the median group

N: The total number of observations

F: The cumulative frequency up to the median group

f: The frequency of the median group

w: The range of the median group

Estimate of standard deviation (σ) = $\sqrt{\sum n_i (m_i - \mu)^2 /_{N-1}}$

where:

n_i: The frequency of the *i* th bin

m_i: The midpoint of the *i* th bin

μ: The mean

N: The total sample size
