# Supplementary material for: Preparing early economic evaluations for the development and management of health service interventions
Source: Int J Technol Assess Health Care. 2024 Oct 18;40(1):e47. doi: 10.1017/S0266462324000539 (PMC11563182; doi:10.1017/S0266462324000539)
Supplement: Partington et al. supplementary material [file S0266462324000539sup001.zip › Appendix 4 SEE interview topic guide - LOS.pdf]

<u>Thanks for your participation</u>

My name is [ ], and I am a part of the research team at Flinders University.

Thanks for agreeing to be interviewed.

Your participation is completely voluntary. Replying to our invitation email and acceptance of our invitation suggest written consent to take part in the study. If you change your mind, you can withdraw at any time and you do not have to give a reason. If you withdraw your consent, any information you may have already provided us will be deleted.

As mentioned in the information sheet, we would like you to give us your expert opinion on a few quantities, for which no empirical data is available. We will ask this information from you in several ways. You will be given a chance to adjust your answers and to provide feedback about the exercise.

We are also asking that, as much as possible, you ‘think aloud’ as we progress through the questions. We ask this because we’re also wanting to understand your thought process and reasoning behind your responses.

Please feel free to ask me any question at any time during the interview. You may also contact the Flinders Health and Medical Research Institute (FHMRI) following the interview if you have any questions related to the study.

I will now turn on the tape-recorder (or Zoom/MS Teams recorder) and ask you a series of questions about the CARE service initiative. Please feel free to turn your video-off and follow along with the Mural whiteboard. Before we begin though, can you confirm whether you consent to being recorded?

[Start recording]

Expected ***future*** values, based on evidence, experience, and judgements

1. *Judgement by* ***anchoring***

… fixing your response to a question, based on a value provided earlier

2. *Judgement by* ***availability***

… attributing a higher probability to events that are familiar

3. *Being* ***overconfident***

… having a too narrow range of possibilities

ED LOS (Hours), 2020

Patient Flow with Existing Care → Present to ED → Not Admitted / Admitted → Disarched from EECU / Receive ward-based care

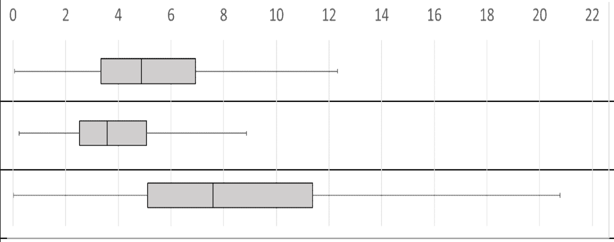

	Volume	Observed Min	Q1	Ave	SD	Median	Q3	Observed Max
Not Admitted	2232	0.07	3.3	**5.6**	3.5	4.9	6.9	23.3
Admitted – Disarched from EECU	2042	0.23	2.5	**4.1**	2.4	3.6	5.1	28.2
Admitted – Receive ward-based care	6556	0.02	5.1	**9.3**	25.7	7.6	11.4	1434.1

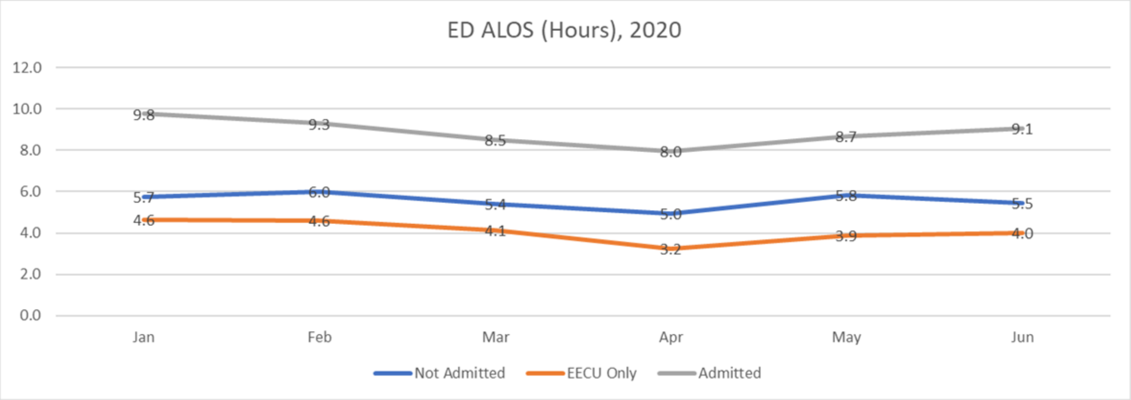

Inpatient LOS (Days), 2020

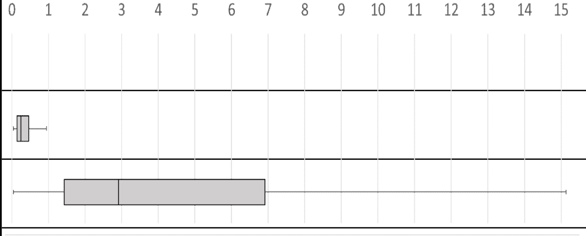

Patient Flow with Existing Care				Volume	Observed Min	Min Outlier Range	Q1	Ave	SD	Median	Q3	Max Outlier Range	Observed Max
	Present to ED	Not Admitted		2232									
		Admitted	Disarched from EECU	2042	0.0	-0.3	0.1	**0.3**	0.3	0.2	0.5	0.9	2.8
			Receive ward-based care	6556	0.0	-6.8	1.4	**6.8**	10.8	2.9	6.9	15.1	160.5

N of admitted patients 6556

Service Types	ALOS for Total Admitted Popn	Sub-popn of those who receive service type		ALOS for those who receive
		n	%	
los_CAP	0.3	118	1.8%	16.1
los_CCU	0.2	402	6.1%	2.7
los_DIAL	0.0	66	1.0%	0.6
los_EECU	0.3	6286	95.9%	0.3
los_FPH	0.0	12	0.2%	0.5
los_FPHAng	0.0	220	3.4%	0.1
los_GEM	0.7	308	4.7%	15.5
los_HDU	0.1	286	4.4%	2.1
los_HITH	0.2	202	3.1%	7.1
los_ICU	0.1	196	3.0%	3.3
los_MH	0.0	18	0.3%	14.2
los_NEON	0.0	2	0.0%	62.6
los_OST	0.0	26	0.4%	1.8
los_PAED	0.0	0	0.0%	-
los_PALL	0.1	54	0.8%	6.6
los_REHAB	0.7	212	3.2%	22.7
los_RITH	0.1	88	1.3%	10.9
los_RV	0.2	70	1.1%	16.8
los_TOW	3.8	5904	90.1%	4.2
AVERAGE	6.8			

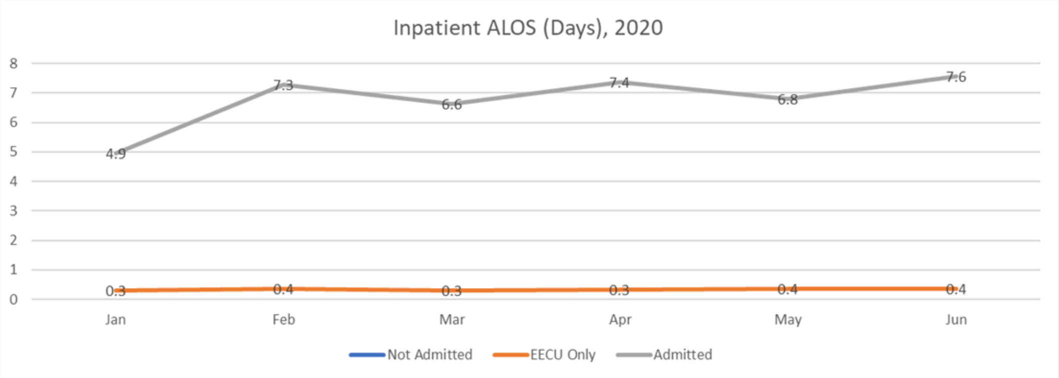

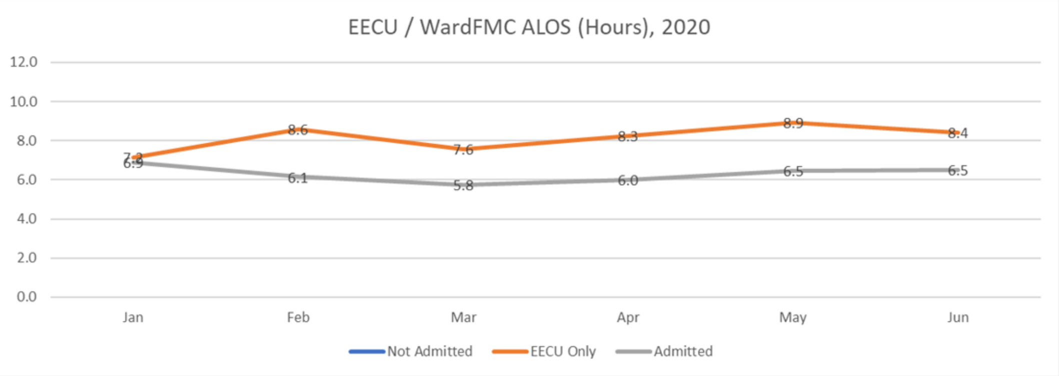

7 Hours ALOS in CARE (min: 0.08 hours, max: 9 hours)

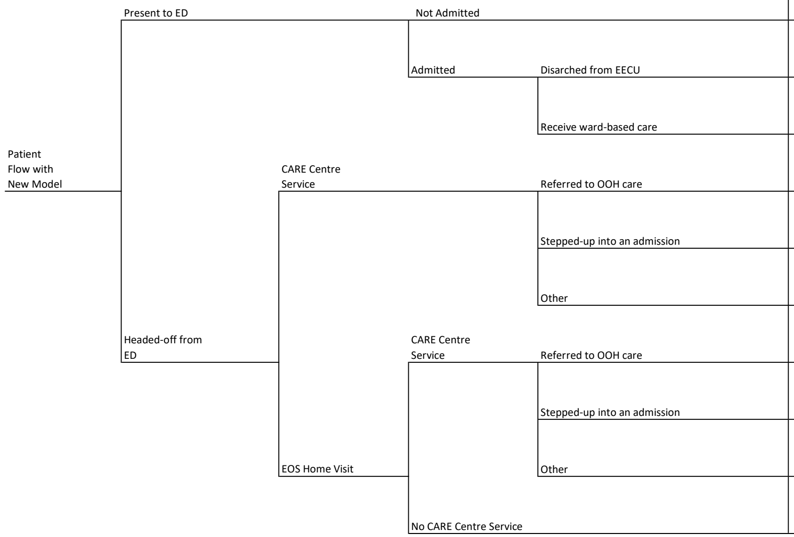

Pathway Number	Pathway Description	Average Length of Stay (ALOS)								
		ED (Hours)			Inpatient (Days)			CARE &/or EOS Service (Hours)		
		Lowest	*Best*	*Highest*	*Lowest*	*Best*	*Highest*	*Lowest*	*Best*	*Highest*
1	Those who continue to present to the ED (i.e., not headed-off), but who are not admitted									
2	Those who continue to present to the ED (i.e., not headed-off), and admitted but discharged from the EECU									
3	Those who continue to present to the ED (i.e., not headed-off), admitted and receive ward-based care									
4	Those who are headed-off from the ED, and referred to further OOH care, via the CARE centre and having first had a home visit from the EOS team									
5	Those who are headed-off from the ED, and who are stepped-up into an admission, via the CARE centre									
6	Those who are headed-off from the ED, and follow other pathways, via the CARE centre									
7	Those who are headed-off from the ED, and referred to further OOH care, via the CARE centre and having first had a home visit from the EOS team									
8	Those who are headed-off from the ED, and who are stepped-up into an admission, via the CARE centre and having first had a home visit from the EOS team									
9	Those who are headed-off from the ED, and follow other pathways, via the CARE centre and having first had a home visit from the EOS team									
10	Those who are headed-off from the ED and do not visit the CARE centre after having a home visit from the EOS team									

I would like to ask you a few questions about your experience with these types of statistical methods

C1. Have you participated before in exercises that formally capture expert opinion?

C2. What type of exercises?

C3. Would you consider your level of statistical knowledge to be:

**Poor**	**Limited**	**Fair**	**Good**	**Excellent**

C4. What potential conflicts of interest might you have that could be perceived as influencing your responses today?

F1.A Please consider how difficult it was for you to complete this exercise.

On a scale from 1 to 5, where 1 means "extremely difficult to complete" and 5 means "extremely easy to complete", how difficult did you consider this exercise[illegible]

F1.B Please think about the difficulty of this exercise.

Can you indicate any specific obstacles you encountered in completing it?

F2.A Now consider how faithfully this exercise has captured your belief about the elicited value.

On a scale from 1 to 5, where 1 means "not at all faithful" and 5 means "exactly as I believe", how would you rate the face validity of this exercise?

F2.B Now please think about the "face validity" of this task (how successfully have we recorded your exact belief).

Can you indicate any specific obstacles that prevented the faithful capture of your belief?

F3.A Do you have any questions about the session we had today?

CLOSURE

Thank you very much for taking part in this study.
