# Supplementary material for: Preparing early economic evaluations for the development and management of health service interventions
Source: Int J Technol Assess Health Care. 2024 Oct 18;40(1):e47. doi: 10.1017/S0266462324000539 (PMC11563182; doi:10.1017/S0266462324000539)
Supplement: Partington et al. supplementary material [file S0266462324000539sup001.zip › Appendix 5 Review and Revise Elicited Estimates.pdf]

# Respondent: R1

Welcome to the opportunity to revise, compare and potentially update your previously provided estimates for patient pathways and lengths of stay associated with the Complex And RestorativE (CARE) service.

The figures display the range of responses provided by yourself and the other respondants. They are numbered (R1) to (R5), with (P) an estiamate that is pooled from the reponses provided.
A space is provided for you on the right-hand side, to view your original estimates and update these. Those cells couloured peach are the only cells that can be edited.

## Pathway proportions

**1** **Of those who would otherwise present to the ED, but would not be admitted**

What % or proportion would you expect to be 'headed-off from the ED' *via the Care Centre* ?

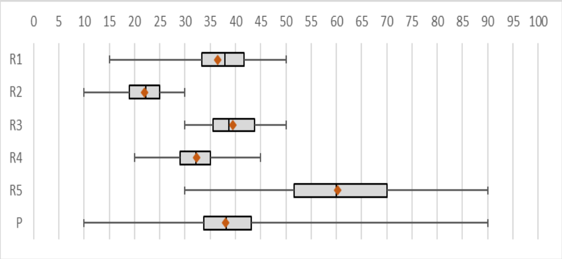


| | Minimum possible | Most likely (average) | Maximum possible |
|---|---|---|---|
| Your original estimates: | 15 | 37 | 50 |
| Updated estimates? | | | |

***Themes of toughts expressed aloud by respondents during the exercise:***

| Patient group | Existing care context | Early trialling of intervention | Adaptations to intervention | External factors and confounders |
|---|---|---|---|---|
| • In practice, the CARE population is an older subset of the pre-specified 65yo+ target population (~80yo).<br>• Often those who would be in 'grey zone' between needing short admission vs. could be discharged from ED.<br>• The older and more fragile you get, the more likely you are to get admitted – not for long, but still get admitted. | • If not given an alternative, all CARE patients will present to an ED. | • We have some personal experience in watching the numbers.<br>• Of 10 people in the CARE centre, about 1/3 are admitted, so they're clearly in the CARE stream.<br>• I would have hoped we can capture most if not all of those presenting to the ED who are discharged home with simple presenting complaints and issues. | | |

**2** **Of those who would otherwise present to the ED, but would not be admitted**

What % or proportion would you expect to 'headed-off from the ED' *via an Eyes On Scene home visit* ?

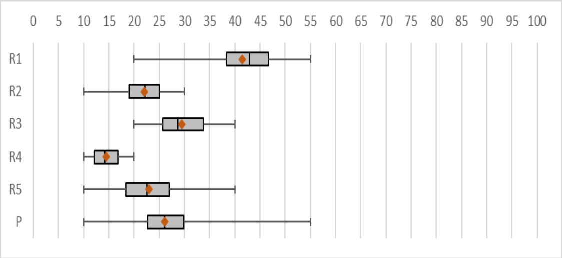


| | Minimum possible | Most likely (average) | Maximum possible |
|---|---|---|---|
| Your original estimates: | 20 | 42 | 55 |
| Updated estimates? | | | |

***Themes of toughts expressed aloud by respondents during the exercise:***

| Patient group | Existing care context | Early trialling of intervention | Adaptations to intervention | External factors and confounders |
|---|---|---|---|---|
| • Compared to the physical CARE Centre, less of those who are otherwise being discharged home from ED will flow to Eyes On Scene, because it's a higher risk approach.<br>• Thought is that Eyes On Seen patients are probably from the 'otherwise admitted' stream, due to the fact that they're coming from residential aged care and they tend to get admitted if presenting to ED.<br>• Common reason for referral to Eyes On Scene is that people's behaviour is deteriorating. | • If CARE doesn't exist, nursing homes will call an ambulance.<br>• If not given an option, these people will present to ED. | • Nursing homes call CARE directly, otherwise EOS receive calls from the Ambulance to attend nursing homes. | | |

**3** **Of those who would otherwise be admitted through the ED, but discharged from the EECU**

What % or proportion would you expect to be 'headed-off from the ED' *via the Care Centre* ?

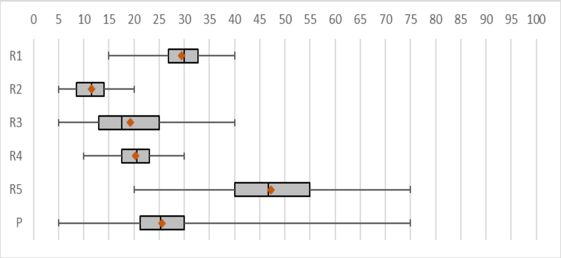


| | Minimum possible | Most likely (average) | Maximum possible |
|---|---|---|---|
| Your original estimates: | 15 | 30 | 40 |
| Updated estimates? | | | |

***Themes of toughts expressed aloud by respondents during the exercise:***

| Patient group | Existing care context | Early trialling of intervention | Adaptations to intervention | External factors and confounders |
|---|---|---|---|---|
| | • The way we use the EECU keeps changing, and varies from consultant to consultant in the ED. | • A feature of CARE is that when patients come into the Centre, we can then follow them up with an Eyes On Scene home visit the next day. | | |

**4** **Of those who would otherwise be admitted through the ED, but discharged from the EECU**

What % or proportion would you expect to 'headed-off from the ED' *via an Eyes On Scene home visit* ?

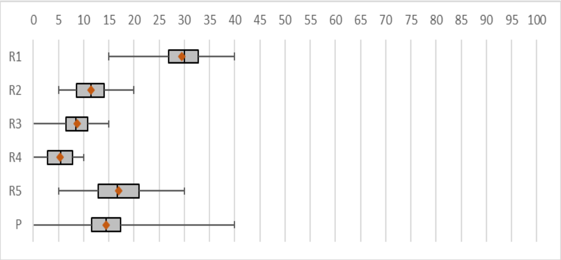


| | Minimum possible | Most likely (average) | Maximum possible |
|---|---|---|---|
| Your original estimates: | 15 | 30 | 40 |
| Updated estimates? | | | |

***Themes of toughts expressed aloud by respondents during the exercise:***

| Patient group | Existing care context | Early trialling of intervention | Adaptations to intervention | External factors and confounders |
|---|---|---|---|---|
| | • Patients presenting to ED in the mid-to-late evening tend to be kept/admitted in EECU until a geriatrician can see them the next morning.<br>• Anecdotally, rather than hanging on to them, ED teams move patients into an admitted space from which they're then moved home the next day.<br>• Admitted care is often arranged ED work from the previous day. | | | |

**5** **Of those who would otherwise be admitted through the ED, and spend time on a ward**

What % or proportion would you expect to be 'headed-off from the ED' *via the Care Centre* ?

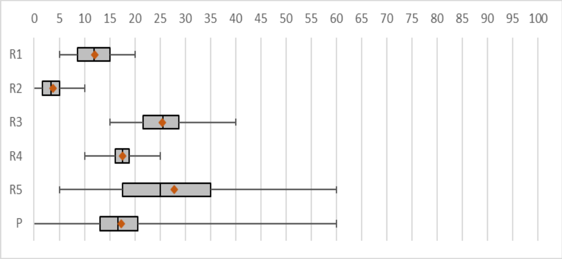


| | Minimum possible | Most likely (average) | Maximum possible |
|---|---|---|---|
| Your original estimates: | 5 | 12 | 20 |
| Updated estimates? | | | |

***Themes of toughts expressed aloud by respondents during the exercise:***

| Patient group | Existing care context | Early trialling of intervention | Adaptations to intervention | External factors and confounders |
|---|---|---|---|---|
| • Includes those who need an x-ray for potential broken hip or brain scan for bleed after a fall. Specifically, those at suspected low of surgery being required.<br>• Under-triaging is a risk. There are instances of patients who would not have been accepted to CARE, had the whole story been known. | | | | |

**6** **Of those who would otherwise be admitted through the ED, and spend time on a ward**

What % or proportion would you expect to 'headed-off from the ED' *via an Eyes On Scene home visit* ?

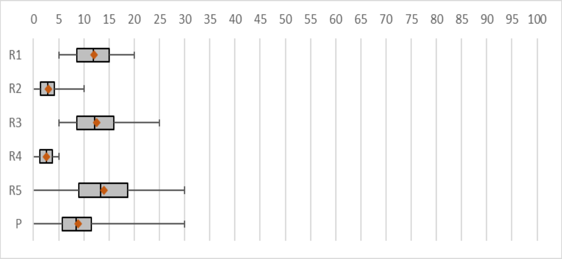


| | Minimum possible | Most likely (average) | Maximum possible |
|---|---|---|---|
| Your original estimates: | 5 | 12 | 20 |
| Updated estimates? | | | |

***Themes of toughts expressed aloud by respondents during the exercise:***

| Patient group | Existing care context | Early trialling of intervention | Adaptations to intervention | External factors and confounders |
|---|---|---|---|---|
| | | | • Were originally only taking from the Ambulance service, but Eyes on Scene visits can now be triggered from a direct call from nursing homes. | |

**7** **Of those who visit the CARE centre**

What % or proportion would you expect to be referred on to Out-Of-Hospital care i.e., GEM@Home?

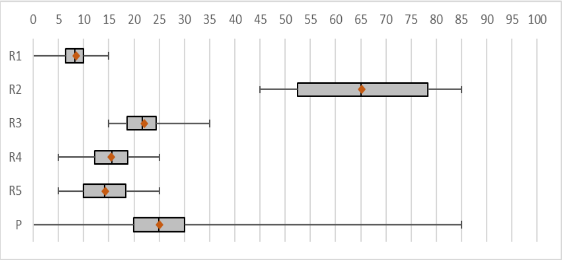


| | Minimum possible | Most likely (average) | Maximum possible |
|---|---|---|---|
| Your original estimates: | 0 | 9 | 15 |
| Updated estimates? | | | |

***Themes of toughts expressed aloud by respondents during the exercise:***

| Patient group | Existing care context | Early trialling of intervention | Adaptations to intervention | External factors and confounders |
|---|---|---|---|---|
| | • Complimentary services like GEM@Home is often a good option, but there may be a two or three day wait. So referrers look for other alternatives. | • Existing low rates of onward referral may reflect capacity issues (e.g., in GEM@Home).<br>• If we incorporate all potential services for onward referral, then the rate may be higher. | • For the first few weeks and months of CARE starting, GEM@Home and other onwards referral services didn't exist.<br>• Referrers are becoming braver in terms of picking up patients who are frailer and would have otherwise been taken further into the hospital. | |

**8** **Of those who visit the CARE centre**

What % or proportion would you expect to be stepped-up into a hospital presentation?

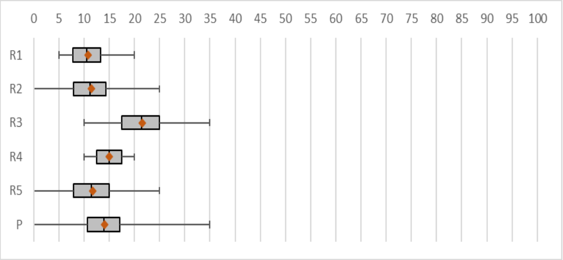


| | Minimum possible | Most likely (average) | Maximum possible |
|---|---|---|---|
| Your original estimates: | 5 | 11 | 20 |
| Updated estimates? | | | |

***Themes of toughts expressed aloud by respondents during the exercise:***

| Patient group | Existing care context | Early trialling of intervention | Adaptations to intervention | External factors and confounders |
|---|---|---|---|---|
| | | • Some days involve no transfers, and others involve multiple. | | |

**9** **Of those who receive an Eyes on Scene home visit**

What % or proportion would you expect to subsequently visit the CARE centre?

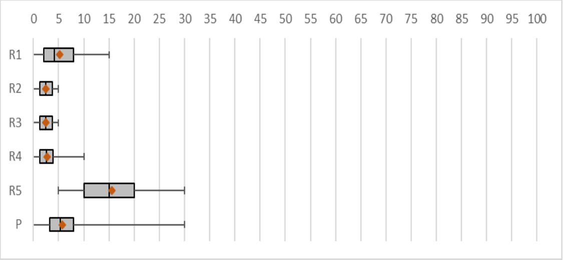


| | Minimum possible | Most likely (average) | Maximum possible |
|---|---|---|---|
| Your original estimates: | 0 | 5 | 15 |
| Updated estimates? | | | |

***Themes of toughts expressed aloud by respondents during the exercise:***

| Patient group | Existing care context | Early trialling of intervention | Adaptations to intervention | External factors and confounders |
|---|---|---|---|---|
| | | • May not involve urgent transfers on the day of CARE, but more routine assessment. | • Rates of transfers might fluctuate if there are increases in workforce demand and needing to deploy less experienced nurses. There are tensions here.<br>• A majority of Eyes On Scene visits are now a follow-up from a CARE centre visit. | |

**10** **Of those who receive an Eyes on Scene home visit**

What % or proportion would you expect to be stepped-up into a hospital presentation?

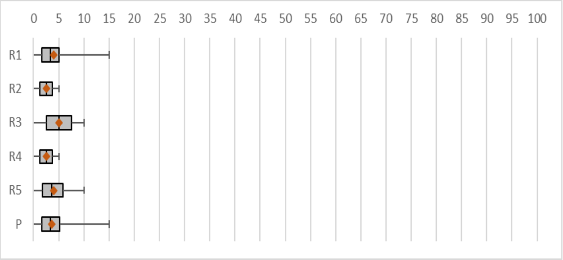


| | Minimum possible | Most likely (average) | Maximum possible |
|---|---|---|---|
| Your original estimates: | 0 | 4 | 15 |
| Updated estimates? | | | |

***Themes of toughts expressed aloud by respondents during the exercise:***

| Patient group | Existing care context | Early trialling of intervention | Adaptations to intervention | External factors and confounders |
|---|---|---|---|---|
| • The hope is that patients who receive Eyes On Scene as an index visit, is part of a very highly selective process. | | • If the Eyes On Scene visit was a follow-up, then that is a bit more likely, but even then is preferred that they come back to the CARE centre.<br>• Early experience is that it is very rare, but that it has happened. | | |

**11** **Of those who visit the CARE centre, following a home visit**

What % or proportion would you expect to be referred on to Out-Of-Hospital care i.e., GEM@Home?

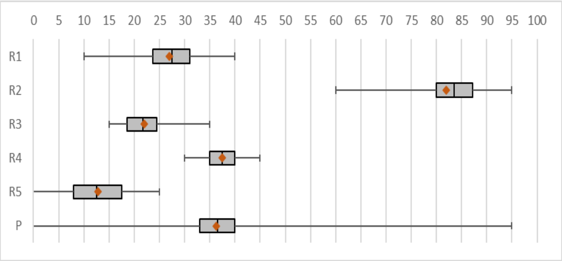


| | Minimum possible | Most likely (average) | Maximum possible |
|---|---|---|---|
| Your original estimates: | 10 | 27 | 40 |
| Updated estimates? | | | |

***Themes of toughts expressed aloud by respondents during the exercise:***

| Patient group | Existing care context | Early trialling of intervention | Adaptations to intervention | External factors and confounders |
|---|---|---|---|---|
| • There is a multiple sifting process here for patient selection in this group.<br>• If needing to come to CARE following an Eyes On Scene, the patients may be a more complex group who we're trying really hard to keep out of the hospital.<br>• Sometimes bring someone into CARE centre because they require imaging. It may not necessarily translate to higher up transfer rates, relative to those who come straight to the CARE centre. | | | • We should not forget that people can be discharged to a new aged care facility, rather than returning to their won current residence. This is particularly relevant for those on respite, while looking for a nursing home, and who may be running out of respite days. | |

**12** **Of those who visit the CARE centre, following a home visit**

What % or proportion would you expect to be stepped-up into a hospital presentation?

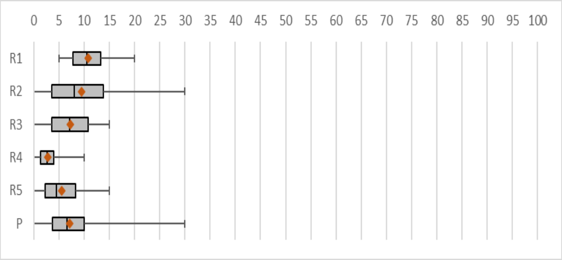


| | Minimum possible | Most likely (average) | Maximum possible |
|---|---|---|---|
| Your original estimates: | 5 | 11 | 20 |
| Updated estimates? | | | |

***Themes of toughts expressed aloud by respondents during the exercise:***

| Patient group | Existing care context | Early trialling of intervention | Adaptations to intervention | External factors and confounders |
|---|---|---|---|---|
| • There is at least some change that patients come into the CARE centre for an x-ray and there is a significant fracture requiring hospitalisation, for example. But it is such a small number of people, given they have already been triaged and received care. | | | | |

# Average Length of Stay/Service

** Please note - the range of hours and days on the x-axis can change between each graph **

## Emergency Department (Hours)

**13** **For those who continue to present to the ED (i.e., not headed-off), but who are not admitted**

What Average Length of Stay (ALOS) would you expect?

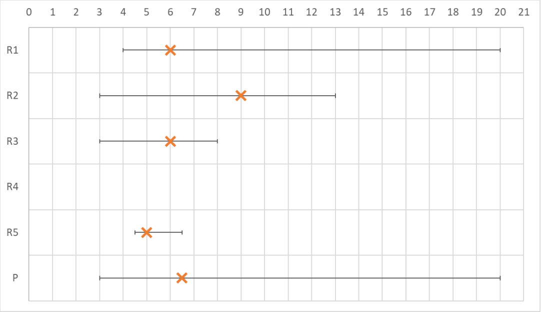


| | Lowest Estimate | Best Estimate | Highest Estimate |
|---|---|---|---|
| Your original estimates: | 4.0 | 6.0 | 20.0 |
| Updated estimates? | | | |

***Themes of toughts expressed aloud by respondents during the exercise:***

| Patient group | Existing care context | Early trialling of intervention | Adaptations to intervention | External factors and confounders |
|---|---|---|---|---|
| | • CARE-type patients may repeatedly be put at the end of a queue, behind trauma patients. This is what takes so long. It doesn't take as long as is seen in the data, to assess what is wrong.<br>• High level trauma patients are processed quickly, and having relatively more of these patients can considerably lower average LOS over a period.<br>• Patients can wait a long time in the ED for imaging. | | | • There are other capacity issues that impact on ALOS.<br>• ALOS is also dependent on whether more/less patients arrive during the day/night. |

**14** **Those who continue to present to the ED (i.e., not headed-off), and admitted but discharged from the EECU**

What Average Length of Stay (ALOS) would you expect?

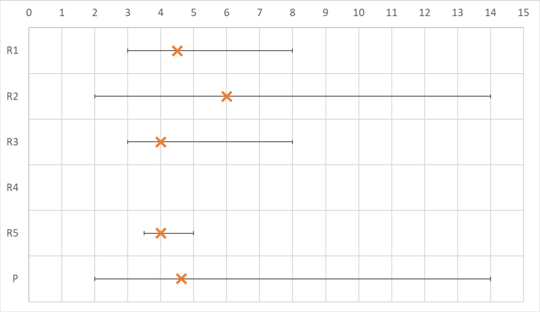


| | Lowest Estimate | Best Estimate | Highest Estimate |
|---|---|---|---|
| Your original estimates: | 3.0 | 4.5 | 8.0 |
| Updated estimates? | | | |

***Themes of toughts expressed aloud by respondents during the exercise:***

| Patient group | Existing care context | Early trialling of intervention | Adaptations to intervention | External factors and confounders |
|---|---|---|---|---|
| | • There is a minimum number of hours required to get done what needs to get done e.g., turn-over rates for results of testing.<br>• ED LOS is slightly shorter for those going to EECU because ED teams can identify earlier that they wouldn't be able to discharge immediately anyway, and so the decision to admit happens relatively quickly. | • If there are LOS changes in the ED, it is because of something that they have changed independently of CARE. | | |

**15** **Those who continue to present to the ED (i.e., not headed-off), admitted and receive ward-based care**

What Average Length of Stay (ALOS) would you expect?

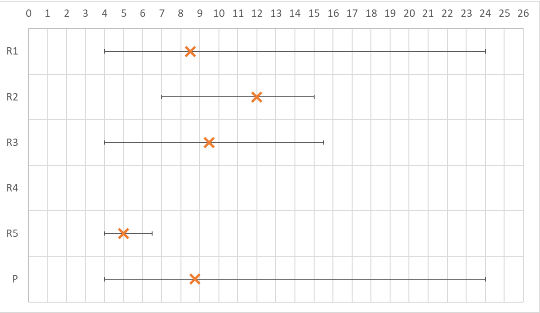


| | Lowest Estimate | Best Estimate | Highest Estimate |
|---|---|---|---|
| Your original estimates: | 4.0 | 8.5 | 24.0 |
| Updated estimates? | | | |

***Themes of toughts expressed aloud by respondents during the exercise:***

| Patient group | Existing care context | Early trialling of intervention | Adaptations to intervention | External factors and confounders |
|---|---|---|---|---|
| | | | | |

## CARE &/or EOS Service (Hours)

**16** **Those who are headed-off from the ED, via the CARE centre**

What Average Length of Stay (ALOS) would you expect?

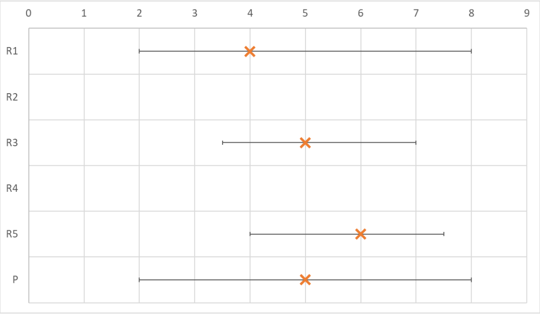


| | Lowest Estimate | Best Estimate | Highest Estimate |
|---|---|---|---|
| Your original estimates: | 2.0 | 4.0 | 8.0 |
| Updated estimates? | | | |

***Themes of toughts expressed aloud by respondents during the exercise:***

| Patient group | Existing care context | Early trialling of intervention | Adaptations to intervention | External factors and confounders |
|---|---|---|---|---|
| | | • A factor is the turn-around of blood results, which are not processed at the CARE centre site. | • Portable/mobile blood testing equipment may soon be available, which would minimise the need to formally send blood offsite. | |

**17** **Those who are headed-off from the ED via the CARE centre, and having first had a home visit from the EOS team**

What Average Length of Stay (ALOS) would you expect?

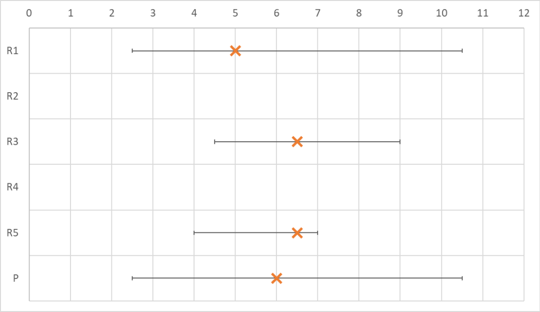


| | Lowest Estimate | Best Estimate | Highest Estimate |
|---|---|---|---|
| Your original estimates: | 2.5 | 5.0 | 10.5 |
| Updated estimates? | | | |

***Themes of toughts expressed aloud by respondents during the exercise:***

| Patient group | Existing care context | Early trialling of intervention | Adaptations to intervention | External factors and confounders |
|---|---|---|---|---|
| • These are perhaps a marginally frailer and sicker population. | | • While the Eyes On Scene visit may confidently rule out the need for admission, it is sometimes the case that the team want a patient to be assessed by a doctor in the CARE centre.<br>• In this case, initial assessments that would otherwise be done in the CARE centre are already conducted during the home visit and we already have that information.<br>• Sometimes patients wait at home between being visited by the Eyes On Scene, and their being retrieved for a CARE centre visit. The wait time may be associated with blood test turn-around. But we're only looking at service time here, not waiting or travelling. | | |

**18** **Those who are headed-off from the ED and do not visit the CARE centre after having a home visit from the EOS team**

What Average Length of Stay (ALOS) would you expect?

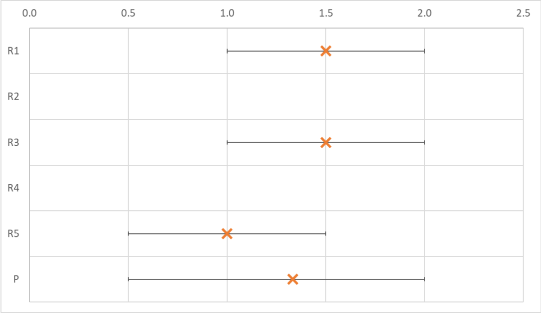


| | Lowest Estimate | Best Estimate | Highest Estimate |
|---|---|---|---|
| Your original estimates: | 1.0 | 1.5 | 2.0 |
| Updated estimates? | | | |

***Themes of toughts expressed aloud by respondents during the exercise:***

| Patient group | Existing care context | Early trialling of intervention | Adaptations to intervention | External factors and confounders |
|---|---|---|---|---|
| | | • This is 'per visit' rather than the cumulative total across multiple home visits. It also only represents the index visit. The cumulative time spent with patients over multiple visits would be higher, but realistically, there is only so much that can be done on site at a home visit. The average time for each of several visits is perhaps similar. | | |

## Inpatient (Days)

**19** **Those who continue to present to the ED (i.e., not headed-off), and admitted but discharged from the EECU**

What Average Length of Stay (ALOS) would you expect?
(Same patient group as in Question 14)

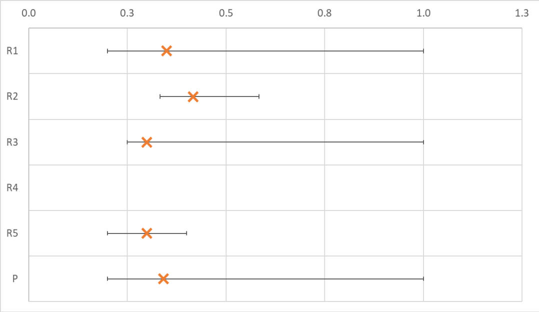


| | Lowest Estimate | Best Estimate | Highest Estimate |
|---|---|---|---|
| Your original estimates: | 0.2 | 0.4 | 1.0 |
| Updated estimates? | | | |

***Themes of toughts expressed aloud by respondents during the exercise:***

| Patient group | Existing care context | Early trialling of intervention | Adaptations to intervention | External factors and confounders |
|---|---|---|---|---|
| • The EECU ALOS includes patients who are in overnight for observations and sometimes because they presented to the ED in the evening. | | | | |

**20** **Those who continue to present to the ED (i.e., not headed-off), admitted and receive ward-based care**

What Average Length of Stay (ALOS) would you expect?
(Same patient group as in Question 15)

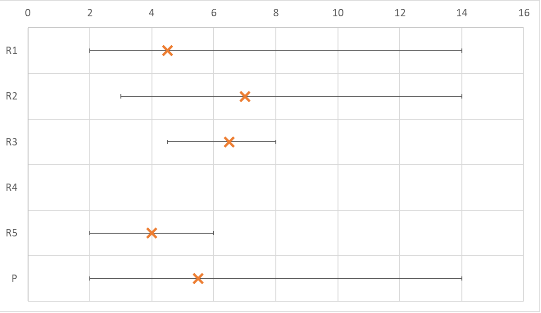


| | Lowest Estimate | Best Estimate | Highest Estimate |
|---|---|---|---|
| Your original estimates: | 2.0 | 4.5 | 14.0 |
| Updated estimates? | | | |

***Themes of toughts expressed aloud by respondents during the exercise:***

| Patient group | Existing care context | Early trialling of intervention | Adaptations to intervention | External factors and confounders |
|---|---|---|---|---|
| • CARE is probably pulling out some of the 'simpler' admitted patients. | • There is significant inertia in how and how quickly patients flow through the system.<br>• There have been some recent changes to out-of-hospital care, with the addition of alternative services. | • While avoided admissions may not reflect a high number of individuals, it is potentially a significant number of beds (i.e., because of a long LOS). | | |

**21** **Those who are headed-off from the ED via the CARE centre, but stepped-up into admitted care**

What Average Length of Stay (ALOS) would you expect?
(A sub-group of patients asked about in Question 16)

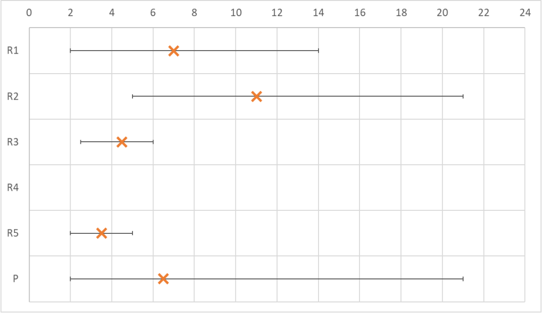


| | Lowest Estimate | Best Estimate | Highest Estimate |
|---|---|---|---|
| Your original estimates: | 2.0 | 7.0 | 14.0 |
| Updated estimates? | | | |

***Themes of toughts expressed aloud by respondents during the exercise:***

| Patient group | Existing care context | Early trialling of intervention | Adaptations to intervention | External factors and confounders |
|---|---|---|---|---|
| | • If patients referred through to emergency respite from CARE were to have been admitted, they could spend two or three weeks in hospital; or 14 days while arranging a nursing home bed.<br>• Conversely, some of the simple patients that attend the CARE centre, may have otherwise been admitted for a few days before going home. | • Early experience of the CARE service indicates around 5% are referred into emergency respite. | | |

**22** **Those who are headed-off from the ED, via the CARE centre and having first had a home visit from the EOS team, but who are stepped-up into an admitted care**

What Average Length of Stay (ALOS) would you expect?
(A sub-group of patients asked about in Question 17)

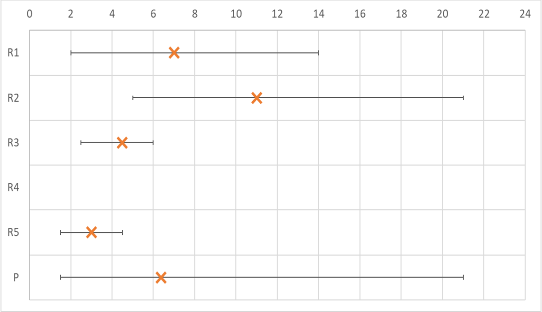


| | Lowest Estimate | Best Estimate | Highest Estimate |
|---|---|---|---|
| Your original estimates: | 2.0 | 7.0 | 14.0 |
| Updated estimates? | | | |

***Themes of toughts expressed aloud by respondents during the exercise:***

| Patient group | Existing care context | Early trialling of intervention | Adaptations to intervention | External factors and confounders |
|---|---|---|---|---|
| • These are a small group, who are not those who come straight for an x-ray and return to the nursing home. | | | | |
