# Supplementary material for: Preparing early economic evaluations for the development and management of health service interventions
Source: Int J Technol Assess Health Care. 2024 Oct 18;40(1):e47. doi: 10.1017/S0266462324000539 (PMC11563182; doi:10.1017/S0266462324000539)

Pooled Estimates of Expected Effects from the Structured Elicitation Exercise

Pathway proportions

Proportion (%)

Summary of pooled empiric distributions

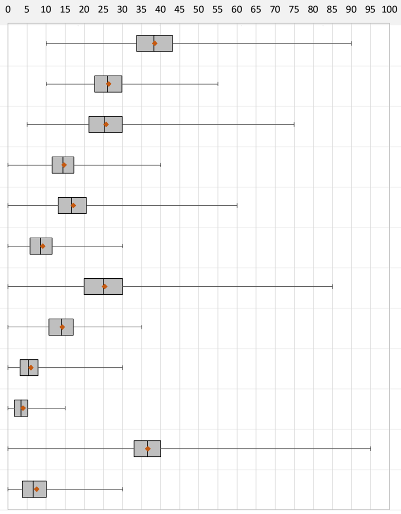

#	Question	Estimates from fitted distributions: Mean (μ)	Std. Dev. (σ)	Fitted parametric distributions: Dist.	α	β	lsq
1	Of those who would otherwise present to the ED, but would not be admitted - What % or proportion would you expect to be 'headed-off from the ED' via the CARE Centre?	38.4	6.9	Beta	18.973	30.390	2.72E-05
2	Of those who would otherwise present to the ED, but would not be admitted - What % or proportion would you expect to be 'headed-off from ED' via an Eyes On Scene home visit?	26.4	5.3	Beta	18.023	50.336	1.17E-05
3	Of those who would otherwise be admitted through the ED, but discharged from the EECU - What % or proportion would you expect to be 'headed-off from the ED' via the CARE Centre?	25.7	6.4	Beta	11.585	33.507	6.23E-05
4	Of those who would otherwise be admitted through the ED, but discharged from the EECU - What % or proportion would you expect to be 'headed-off from the ED' via an Eyes On Scene home visit?	14.7	4.3	Beta	9.857	57.355	1.62E-04
5	Of those who would otherwise be admitted through the ED, and spend time on a ward - What % or proportion would you expect to be 'headed-off from the ED' via the CARE Centre?	17.1	5.5	Beta	7.838	37.982	1.60E-05
6	Of those who would otherwise be admitted through the ED, and spend time on a ward - What % or proportion would you expect to be 'headed-off from the ED' via an Eyes On Scene home visit?	9.1	4.4	Beta	3.762	37.682	1.65E-04
7	Of those who visit the CARE centre - What % or proportion would you expect to be referred on to Out-Of-Hospital care e.g., GEM@Home?	25.3	7.4	Beta	8.536	25.194	7.29E-05
8	Of those who visit the CARE centre - What % or proportion would you expect to be stepped-up into a hospital presentation?	14.2	4.7	Beta	7.559	45.560	2.89E-04
9	Of those who receive an Eyes on Scene home visit - What % or proportion would you expect to subsequently visit the CARE centre?	6.0	3.7	Beta	2.390	37.500	1.91E-04
10	Of those who receive an Eyes on Scene home visit - What % or proportion would you expect to be stepped-up into a hospital presentation?	3.9	2.9	Beta	1.708	41.967	9.36E-04
11	Of those who visit the CARE centre, following a home visit - What % or proportion would you expect to be referred on to Out-Of-Hospital care e.g., GEM@Home?	36.6	5.1	Beta	32.029	55.434	9.68E-06
12	Of those who visit the CARE centre, following a home visit - What % or proportion would you expect to be stepped-up into a hospital presentation?	7.5	5.0	Beta	2.008	24.737	1.56E-04

ED Average Length Of Stay

ALOS (Hours)

Summary of pooled empiric distributions

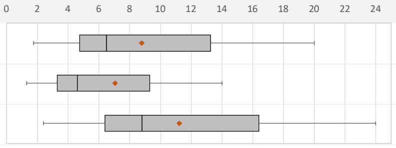

#	Question	Estimates from fitted distributions: Mean (^x)	Std. Dev. (^σ)	Fitted parametric distributions: Dist.	μ	σ	lsq
13	Those who continue to present to the ED (i.e., not headed-off), but who are not admitted	8.8	6.0	Lognormal	1.979	0.623	0.021
14	Those who continue to present to the ED (i.e., not headed-off), and admitted but discharged from the EECU	7.0	6.7	Lognormal	1.633	0.800	0.030
15	Those who continue to present to the ED (i.e., not headed-off), admitted and receive ward-based care	11.2	7.1	Lognormal	2.252	0.577	0.016

CARE &/or EOS Service Average Length Of Stay

ALOS (Hours)

Summary of pooled empiric distributions

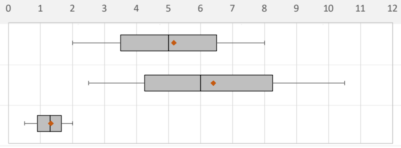

#	Question	Estimates from fitted distributions: Mean (^x)	Std. Dev. (^σ)	Fitted parametric distributions: Dist.	μ	σ	lsq
16	Those who are headed-off from the ED via the CARE centre	5.2	2.1	Lognormal	1.568	0.383	0.014
17	Those who are headed-off from the ED via the CARE centre, and having first had a home visit from the EOS team	6.4	2.8	Lognormal	1.771	0.413	0.010
18	Those who are headed-off from the ED and do not visit the CARE centre after having a home visit from the EOS team	1.3	0.5	Lognormal	0.211	0.373	0.016

Inpatient Average Length Of Stay

ALOS (Days)

Summary of pooled empiric distributions

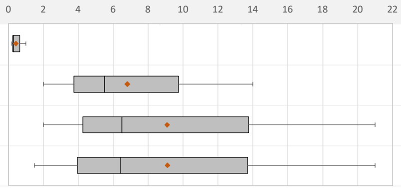

#	Question	Estimates from fitted distributions: Mean (^x)	Std. Dev. (^σ)	Fitted parametric distributions: Dist.	μ	σ	lsq
19	Those who continue to present to the ED (i.e., not headed-off), and admitted but discharged from the EECU	0.4	0.3	Normal	0.426	0.258	0.075
20	Those who continue to present to the ED (i.e., not headed-off), admitted and receive ward-based care	6.8	4.4	Lognormal	1.741	0.595	0.011
21	Those who are headed-off from the ED, and who are stepped-up into an admission, via the CARE centre	9.1	7.6	Lognormal	1.940	0.730	0.013
22	Those who are headed-off from the ED, and who are stepped-up into an admission, via the CARE centre and having first had a home visit from the EOS team	9.1	8.3	Lognormal	1.904	0.780	0.011

Pathway proportions

1 Of those who would otherwise present to the ED, but would not be admitted - What % or proportion would you expect to be 'headed-off from the ED' via the CARE Centre?

Percentage (%)	Respondent					P
	R1	R2	R3	R4	R5	
Min	15.0	10.0	30.0	20.0	30.0	10.0
Q1	33.3	19.0	35.6	29.0	51.7	33.7
Median	37.9	22.2	38.8	32.2	60.0	38.2
Q3	41.7	25.0	43.8	35.0	70.0	43.1
Max	50.0	30.0	50.0	45.0	90.0	90.0
Mean (μ)	36.5	22.0	39.5	32.3	60.3	
Std. Dev. (σ)	7.4	4.3	5.2	4.7	14.4	

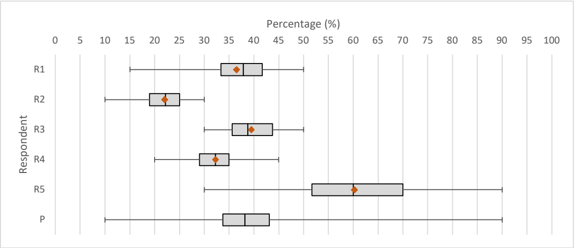

2 Of those who would otherwise present to the ED, but would not be admitted - What % or proportion would you expect to be 'headed-off from ED' via an Eyes On Scene home visit?

Percentage (%)	Respondent					P
	R1	R2	R3	R4	R5	
Min	20.0	10.0	20.0	10.0	10.0	10.0
Q1	38.3	19.0	25.6	12.1	18.3	22.7
Median	42.9	22.2	28.8	14.2	22.5	26.1
Q3	46.7	25.0	33.8	16.9	27.0	29.9
Max	55.0	30.0	40.0	20.0	40.0	55.0
Mean (μ)	41.5	22.0	29.5	14.5	23.0	
Std. Dev. (σ)	7.4	4.3	5.2	2.5	6.0	

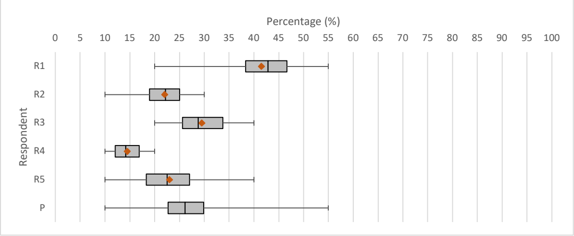

3 Of those who would otherwise be admitted through the ED, but discharged from the EECU - What % or proportion would you expect to be 'headed-off from the ED' via the CARE Centre?

Percentage (%)	Respondent					P
	R1	R2	R3	R4	R5	
Min	15.0	5.0	5.0	10.0	20.0	5.0
Q1	26.9	8.6	13.0	17.5	40.0	21.2
Median	30.0	11.5	17.5	20.5	46.7	25.2
Q3	32.8	14.0	25.0	23.0	55.0	30.0
Max	40.0	20.0	40.0	30.0	75.0	75.0
Mean (μ)	29.5	11.5	19.3	20.3	47.3	
Std. Dev. (σ)	4.4	3.5	8.5	3.4	12.7	

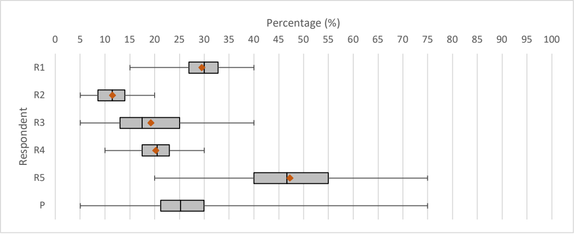

4 Of those who would otherwise be admitted through the ED, but discharged from the EECU - What % or proportion would you expect to be 'headed-off from the ED' via an Eyes On Scene home visit?

Percentage (%)	Respondent					P
	R1	R2	R3	R4	R5	
Min	15.0	5.0	0.0	0.0	5.0	0.0
Q1	26.9	8.6	6.5	2.8	12.9	11.5
Median	30.0	11.5	8.5	5.5	16.7	14.4
Q3	32.8	14.0	10.8	7.7	21.0	17.3
Max	40.0	20.0	15.0	10.0	30.0	40.0
Mean (μ)	29.5	11.5	8.8	5.3	17.0	
Std. Dev. (σ)	4.4	3.5	2.8	2.6	5.1	

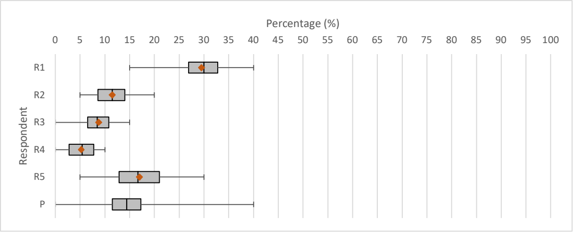

5 Of those who would otherwise be admitted through the ED, and spend time on a ward - What % or proportion would you expect to be 'headed-off from the ED' via the Care Centre?

Percentage (%)	Respondent					P
	R1	R2	R3	R4	R5	
Min	5.0	0.0	15.0	10.0	5.0	0.0
Q1	8.6	1.7	21.7	16.1	17.5	13.1
Median	11.9	3.3	25.6	17.5	25.0	16.7
Q3	15.0	5.0	28.8	18.9	35.0	20.5
Max	20.0	10.0	40.0	25.0	60.0	60.0
Mean (μ)	12.0	3.8	25.5	17.5	27.8	
Std. Dev. (σ)	3.9	2.2	5.2	1.6	13.8	

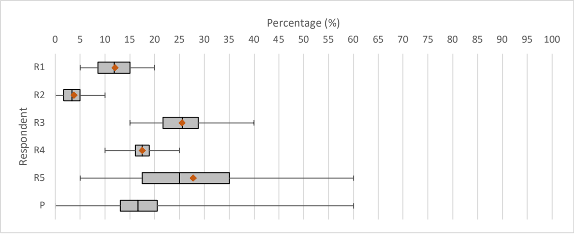

6 Of those who would otherwise be admitted through the ED, and spend time on a ward - What % or proportion would you expect to be 'headed-off from the ED' via an Eyes On Scene home visit?

Percentage (%)	Respondent					P
	R1	R2	R3	R4	R5	
Min	5.0	0.0	5.0	0.0	0.0	0.0
Q1	8.6	1.4	8.6	1.3	9.0	5.8
Median	11.9	2.8	12.1	2.5	13.3	8.5
Q3	15.0	4.2	16.0	3.8	18.8	11.5
Max	20.0	10.0	25.0	5.0	30.0	30.0
Mean (μ)	12.0	3.0	12.5	2.5	14.0	
Std. Dev. (σ)	3.9	1.5	4.6	0.0	6.5	

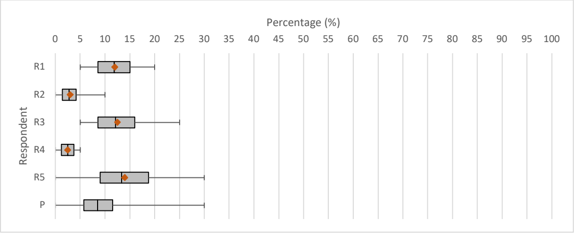

7 Of those who visit the CARE centre - What % or proportion would you expect to be referred on to Out-Of-Hospital care e.g., GEM@Home?

		Respondent					P
		R1	R2	R3	R4	R5	
Percentage (%)	Min	0.0	45.0	15.0	5.0	5.0	0.0
	Q1	6.4	52.5	18.6	12.1	10.0	19.9
	Median	8.2	65.0	21.7	15.6	14.2	24.9
	Q3	10.0	78.3	24.4	18.8	18.3	30.0
	Max	15.0	85.0	35.0	25.0	25.0	85.0
	Mean (μ)	8.5	65.3	22.0	15.5	14.3	
	Std. Dev. (σ)	2.6	13.4	4.3	4.4	5.2	

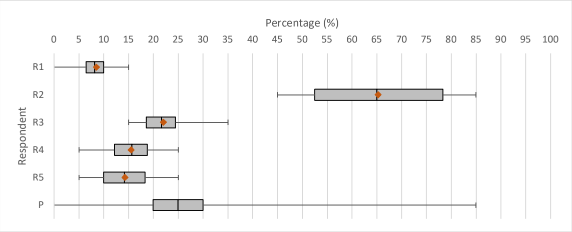

8 Of those who visit the CARE centre - What % or proportion would you expect to be stepped-up into a hospital presentation?

		Respondent					P
		R1	R2	R3	R4	R5	
Percentage (%)	Min	5.0	0.0	10.0	10.0	0.0	0.0
	Q1	7.8	7.9	17.5	12.5	7.9	10.7
	Median	10.6	11.3	21.4	15.0	11.4	13.9
	Q3	13.3	14.4	25.0	17.5	15.0	17.0
	Max	20.0	25.0	35.0	20.0	25.0	35.0
	Mean (μ)	10.8	11.5	21.5	15.0	11.8	
	Std. Dev. (σ)	3.4	4.8	5.3	2.6	4.9	

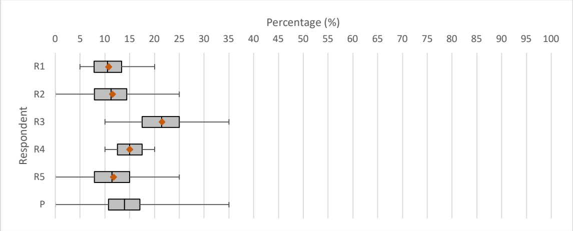

9 Of those who receive an Eyes on Scene home visit - What % or proportion would you expect to subsequently visit the CARE centre?

		Respondent					P
		R1	R2	R3	R4	R5	
Percentage (%)	Min	0.0	0.0	0.0	0.0	5.0	0.0
	Q1	2.1	1.3	1.3	1.3	10.0	3.2
	Median	4.2	2.5	2.5	2.6	15.0	5.4
	Q3	8.0	3.8	3.8	3.9	20.0	7.9
	Max	15.0	5.0	5.0	10.0	30.0	30.0
	Mean (μ)	5.3	2.5	2.5	2.8	15.5	
	Std. Dev. (σ)	3.8	0.0	0.0	1.1	6.6	

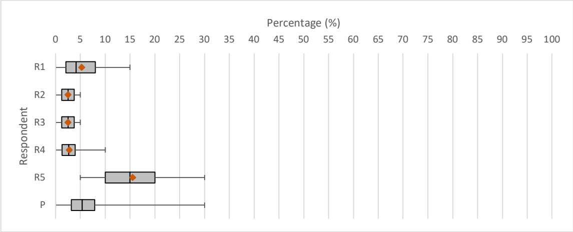

10 Of those who receive an Eyes on Scene home visit - What % or proportion would you expect to be stepped-up into a hospital presentation?

		Respondent					P
		R1	R2	R3	R4	R5	
Percentage (%)	Min	0.0	0.0	0.0	0.0	0.0	0.0
	Q1	1.7	1.3	2.5	1.3	1.8	1.7
	Median	3.3	2.5	5.0	2.5	3.6	3.4
	Q3	5.0	3.8	7.5	3.8	5.8	5.2
	Max	15.0	5.0	10.0	5.0	10.0	15.0
	Mean (μ)	4.0	2.5	5.0	2.5	4.0	
	Std. Dev. (σ)	2.9	0.0	2.6	0.0	2.4	

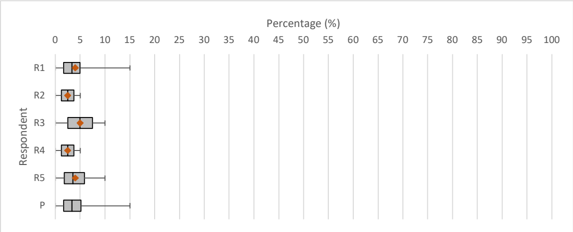

11 Of those who visit the CARE centre, following a home visit - What % or proportion would you expect to be referred on to Out-Of-Hospital care e.g., GEM@Home?

		Respondent					P
		R1	R2	R3	R4	R5	
Percentage (%)	Min	10.0	60.0	15.0	30.0	0.0	0.0
	Q1	23.8	80.0	18.6	35.0	8.0	33.1
	Median	27.5	83.6	21.7	37.5	12.5	36.5
	Q3	31.0	87.1	24.4	40.0	17.5	40.0
	Max	40.0	95.0	35.0	45.0	25.0	95.0
	Mean (μ)	27.0	82.0	22.0	37.5	12.8	
	Std. Dev. (σ)	5.8	7.4	4.3	3.6	6.2	

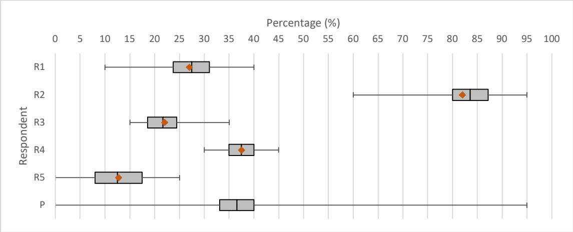

12 Of those who visit the CARE centre, following a home visit - What % or proportion would you expect to be stepped-up into a hospital presentation?

		Respondent					P
		R1	R2	R3	R4	R5	
Percentage (%)	Min	5.0	0.0	0.0	0.0	0.0	0.0
	Q1	7.8	3.6	3.6	1.3	2.3	3.7
	Median	10.6	8.0	7.1	2.6	4.5	6.6
	Q3	13.3	13.8	10.8	3.9	8.3	10.0
	Max	20.0	30.0	15.0	10.0	15.0	30.0
	Mean (μ)	10.8	9.5	7.3	2.8	5.5	
	Std. Dev. (σ)	3.4	7.3	4.1	1.1	3.8	

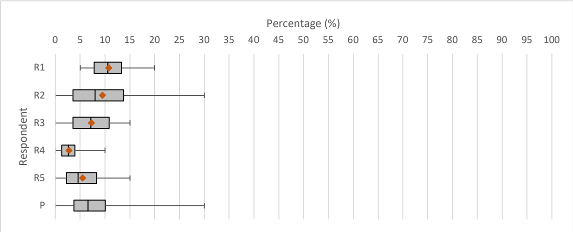

ED ALOS

13 Those who continue to present to the ED (i.e., not headed-off), but who are not admitted

		Respondent					
		R1	R2	R3	R4	R5	P
ALOS (Hours)	Lowest	4.0	3.0	3.0		4.5	3.0
	Best Estimate	6.0	9.0	6.0		5.0	6.5
	Highest	20.0	13.0	8.0		6.5	20.0
	Calc. Q1	*5.0*	*6.0*	*4.5*		*4.8*	*5.1*
	Calc. Q3	*13.0*	*11.0*	*7.0*		*5.8*	*9.2*

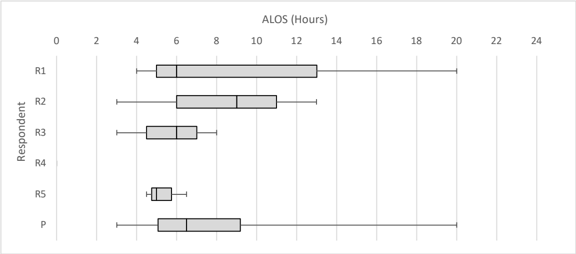

14 Those who continue to present to the ED (i.e., not headed-off), and admitted but discharged from the EECU

		Respondent					
		R1	R2	R3	R4	R5	P
ALOS (Hours)	Lowest	3.0	2.0	3.0		3.5	2.0
	Best Estimate	4.5	6.0	4.0		4.0	4.6
	Highest	8.0	14.0	8.0		5.0	14.0
	Calc. Q1	*3.8*	*4.0*	*3.5*		*3.8*	*3.8*
	Calc. Q3	*6.3*	*10.0*	*6.0*		*4.5*	*6.7*

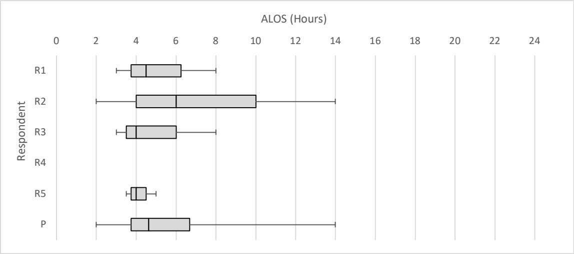

15 Those who continue to present to the ED (i.e., not headed-off), admitted and receive ward-based care

		Respondent					
		R1	R2	R3	R4	R5	P
ALOS (Hours)	Lowest	4.0	7.0	4.0		4.0	4.0
	Best Estimate	8.5	12.0	9.5		5.0	8.8
	Highest	24.0	15.0	15.5		6.5	24.0
	Calc. Q1	*6.3*	*9.5*	*6.8*		*4.5*	*6.8*
	Calc. Q3	*16.3*	*13.5*	*12.5*		*5.8*	*12.0*

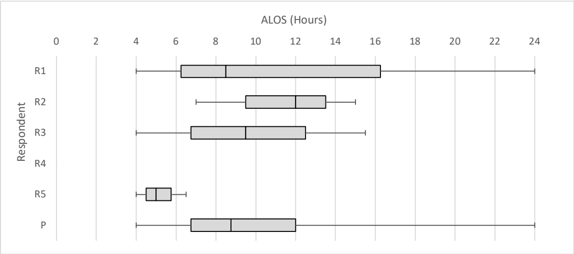

CARE &/or EOS Service ALOS

16 Those who are headed-off from the ED, via the CARE centre

		Respondent					
		R1	R2	R3	R4	R5	P
ALOS (Hours)	Lowest	2.0		3.5		4.0	2.0
	Best Estimate	4.0		5.0		6.0	5.0
	Highest	8.0		7.0		7.5	8.0
	Calc. Q1	*3.0*		*4.3*		*5.0*	*4.1*
	Calc. Q3	*6.0*		*6.0*		*6.8*	*6.3*

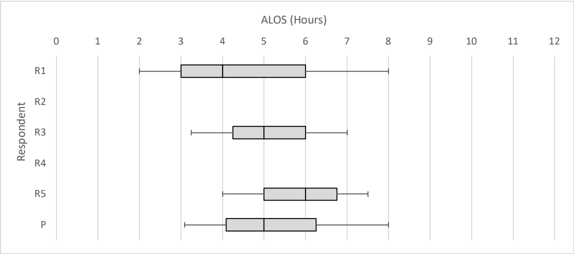

17 Those who are headed-off from the ED via the CARE centre, and having first had a home visit from the EOS team

		Respondent					
		R1	R2	R3	R4	R5	P
ALOS (Hours)	Lowest	2.5		4.5		4.0	2.5
	Best Estimate	5.0		6.5		6.5	6.0
	Highest	10.5		9.0		7.0	10.5
	Calc. Q1	*3.8*		*5.5*		*5.3*	*4.8*
	Calc. Q3	*7.8*		*7.8*		*6.8*	*7.4*

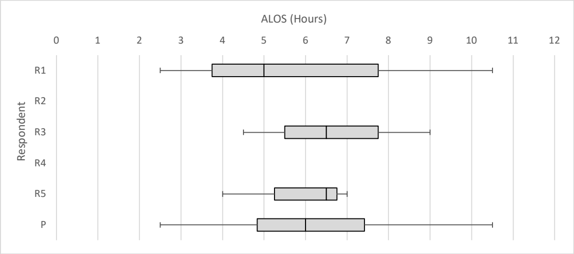

18 Those who are headed-off from the ED and do not visit the CARE centre after having a home visit from the EOS team

		Respondent					
		R1	R2	R3	R4	R5	P
ALOS (Hours)	Lowest	1.0		1.0		0.5	0.5
	Best Estimate	1.5		1.5		1.0	1.3
	Highest	2.0		2.0		1.5	2.0
	Calc. Q1	*1.3*		*1.3*		*0.8*	*1.1*
	Calc. Q3	*1.8*		*1.8*		*1.3*	*1.6*

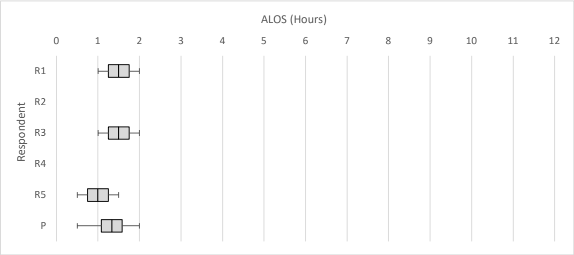

Inpatient ALOS

19 Those who continue to present to the ED (i.e., not headed-off), and admitted but discharged from the EECU

		Respondent					P
		**R1**	**R2**	**R3**	**R4**	**R5**	
**ALOS (Days)**	**Lowest**	0.2	0.3	0.3		0.2	0.2
	**Best Estimate**	0.4	0.4	0.3		0.3	0.3
	**Highest**	1.0	0.6	1.0		0.4	1.0
	Calc. Q1	*0.3*	*0.4*	*0.3*		*0.3*	*0.3*
	Calc. Q3	*0.7*	*0.5*	*0.7*		*0.4*	*0.5*

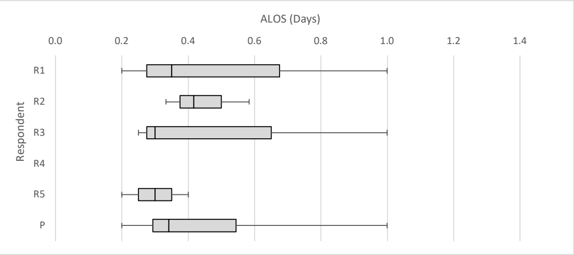

20 Those who continue to present to the ED (i.e., not headed-off), admitted and receive ward-based care

		Respondent					P
		**R1**	**R2**	**R3**	**R4**	**R5**	
**ALOS (Days)**	**Lowest**	2.0	3.0	4.5		2.0	2.0
	**Best Estimate**	4.5	7.0	6.5		4.0	5.5
	**Highest**	14.0	14.0	8.0		6.0	14.0
	Calc. Q1	*3.3*	*5.0*	*5.5*		*3.0*	*4.2*
	Calc. Q3	*9.3*	*10.5*	*7.3*		*5.0*	*8.0*

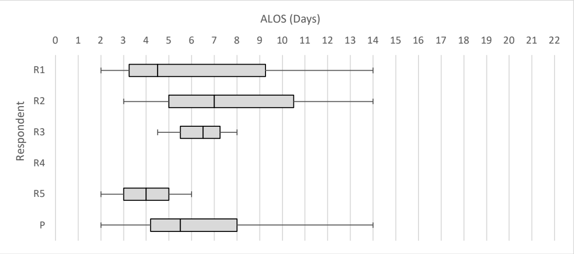

21 Those who are headed-off from the ED, and who are stepped-up into an admission, via the CARE centre

		Respondent					P
		**R1**	**R2**	**R3**	**R4**	**R5**	
**ALOS (Days)**	**Lowest**	2.0	5.0	2.5		2.0	2.0
	**Best Estimate**	7.0	11.0	4.5		3.5	6.5
	**Highest**	14.0	21.0	6.0		5.0	21.0
	Calc. Q1	*4.5*	*8.0*	*3.5*		*2.8*	*4.7*
	Calc. Q3	*10.5*	*16.0*	*5.3*		*4.3*	*9.0*

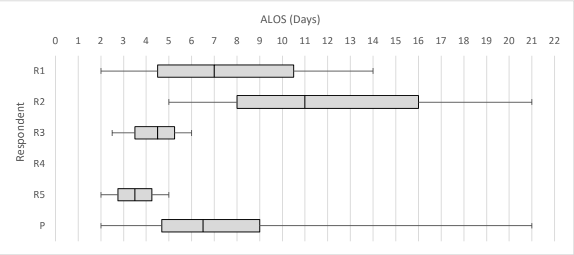

22 Those who are headed-off from the ED, and who are stepped-up into an admission, via the CARE centre and having first had a home visit from the EOS team

		Respondent					P
		**R1**	**R2**	**R3**	**R4**	**R5**	
**ALOS (Days)**	**Lowest**	2.0	5.0	2.5		1.5	1.5
	**Best Estimate**	7.0	11.0	4.5		3.0	6.4
	**Highest**	14.0	21.0	6.0		4.5	21.0
	Calc. Q1	*4.5*	*8.0*	*3.5*		*2.3*	*4.6*
	Calc. Q3	*10.5*	*16.0*	*5.3*		*3.8*	*8.9*

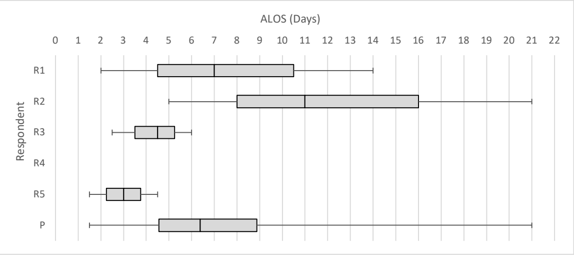

Supplement: Partington et al. supplementary material [file S0266462324000539sup001.zip › Appendix 6 Pooled and Collated SEE results.pdf]
